# Effects of a Telehealth Early Palliative Care Intervention for Family Caregivers of Persons With Advanced Heart Failure: The ENABLE CHF-PC Randomized Clinical Trial

**DOI:** 10.1001/jamanetworkopen.2020.2583

**Published:** 2020-04-13

**Authors:** J. Nicholas Dionne-Odom, Deborah B. Ejem, Rachel Wells, Andres Azuero, Macy L. Stockdill, Konda Keebler, Elizabeth Sockwell, Sheri Tims, Sally Engler, Elizabeth Kvale, Raegan W. Durant, Rodney O. Tucker, Kathryn L. Burgio, Jose Tallaj, Salpy V. Pamboukian, Keith M. Swetz, Marie A. Bakitas

**Affiliations:** 1 University of Alabama at Birmingham School of Nursing; 2 Center for Palliative and Supportive Care, Division of Gerontology, Geriatrics, and Palliative Care, Department of Medicine, University of Alabama at Birmingham; 3 Department of Medicine, Dell Medical School, The University of Texas at Austin; 4 Division of Preventive Medicine, Department of Medicine, University of Alabama at Birmingham; 5 Birmingham Veterans Affairs Medical Center, Birmingham, Alabama; 6 Division of Cardiovascular Diseases, Department of Medicine, University of Alabama at Birmingham

## Abstract

**Importance:**

Family caregivers of persons with advanced heart failure perform numerous daily tasks to assist their relatives and are at high risk for distress and poor quality of life.

**Objective:**

To determine the effect of a nurse-led palliative care telehealth intervention (Educate, Nurture, Advise, Before Life Ends Comprehensive Heart Failure for Patients and Caregivers [ENABLE CHF-PC]) on quality of life and mood of family caregivers of persons with New York Heart Association Class III/IV heart failure over 16 weeks.

**Design, Setting, and Participants:**

This single-blind randomized clinical trial enrolled caregivers aged 18 years and older who self-identified as an unpaid close friend or family member who knew the patient well and who was involved with their day-to-day medical care. Participants were recruited from outpatient heart failure clinics at a large academic tertiary care medical center and a Veterans Affairs medical center from August 2016 to October 2018.

**Intervention:**

Four weekly psychosocial and problem-solving support telephonic sessions lasting between 20 and 60 minutes facilitated by a trained nurse coach plus monthly follow-up for 48 weeks. The usual care group received no additional intervention.

**Main Outcomes and Measures:**

The primary outcomes were quality of life (measured using the Bakas Caregiver Outcomes Scale), mood (anxiety and/or depressive symptoms measured using the Hospital Anxiety and Depression Scale), and burden (measured using the Montgomery-Borgatta Caregiver Burden scales) over 16 weeks. Secondary outcomes were global health (measured using the PROMIS Global Health instrument) and positive aspects of caregiving.

**Results:**

A total of 158 family caregivers were randomized, 82 to the intervention and 76 to usual care. The mean (SD) age was 57.9 (11.6) years, 135 (85.4%) were female, 82 (51.9%) were African American, and 103 (65.2%) were the patient’s spouse or partner. At week 16, the mean (SE) Bakas Caregiver Outcomes Scale score was 66.9 (2.1) in the intervention group and 63.9 (1.7) in the usual care group; over 16 weeks, the mean (SE) Bakas Caregiver Outcomes Scale score improved 0.7 (1.7) points in the intervention group and 1.1 (1.6) points in the usual care group (difference, −0.4; 95% CI, −5.1 to 4.3; Cohen *d* = −0.03). At week 16, no relevant between-group differences were observed between the intervention and usual care groups for the Hospital Anxiety and Depression Scale anxiety measure (mean [SE] improvement from baseline, 0.3 [0.3] vs 0.4 [0.3]; difference, −0.1 [0.5]; *d* = −0.02) or depression measure (mean [SE] improvement from baseline, −0.2 [0.4] vs −0.3 [0.3]; difference, 0.1 [0.5]; *d* = 0.03). No between-group differences were observed in the Montgomery-Borgatta Caregiver Burden scales (*d* range, −0.18 to 0.0). Differences in secondary outcomes were also not significant (*d* range, −0.22 to 0.0).

**Conclusions and Relevance:**

This 2-site randomized clinical trial of a telehealth intervention for family caregivers of patients with advanced heart failure, more than half of whom were African American and most of whom were not distressed at baseline, did not demonstrate clinically better quality of life, mood, or burden compared with usual care over 16 weeks. Future interventions should target distressed caregivers and assess caregiver effects on patient outcomes.

**Trial Registration:**

ClinicalTrials.gov Identifier: NCT02505425

## Key Points

**Question:**

What is the impact of a telehealth early palliative care intervention compared with usual care on the quality of life, mood, and burden of family caregivers of persons with advanced heart failure over 16 weeks?

**Findings:**

In this randomized clinical trial that included 158 family caregivers, half of whom were African American and most of whom were not distressed at baseline, there were no significant differences in primary outcomes over 16 weeks.

**Meaning:**

An early palliative care intervention was not significantly better than usual care at improving the quality of life, mood, and burden of family caregivers of patients with advanced heart failure.

## Teaser

This randomized clinical trial investigates the effect of a nurse-led palliative care telehealth intervention on quality of life and mood of family caregivers of individuals with advanced heart failure.

## Introduction

Most of the more than 6.5 million US individuals with heart failure rely on the regular support of close family members and friends to help maintain their health and quality of life.^1,2^ These heart failure family caregivers assist with numerous health care tasks that have been valued at $7.9 billion in the US.^3^ These tasks include medication and device management, symptom monitoring and management, care coordination and transportation, emotional and informational support, self-care promotion (eg, special diet and meal preparation, physical activity) and decision support.^2,4,5^ Performing these tasks, often with no training and while also coping with seeing a close relative deal with a life-threatening illness, has been associated with high rates of caregiver physical and psychological distress and low quality of life.^2,4,6,7^ This is a critical issue for health care clinicians and hospital systems because unmet needs of family caregivers for patients with heart failure have been associated with more patient distress, poorer patient self-care and medical treatment adherence, and higher readmission rates.^8,9^ To date, few interventions have been developed and tested to address this need.^2,10,11^

In light of this, we developed a nurse-led palliative care intervention for family caregivers of persons with advanced heart failure called ENABLE CHF-PC (Educate, Nurture, Advise, Before Life Ends Comprehensive Heart Failure for Patients and Caregivers), consisting of 4 weekly 20- to 60-minute manualized psychosocial and problem-solving support telephonic sessions facilitated by a registered nurse coach and a study team–developed *Charting Your Course–Caregiver* guidebook plus monthly follow-up for 48 weeks. After performing formative evaluation work to adapt the ENABLE caregiver intervention from cancer to heart failure and refine the intervention in 2 single-group pilot trials,^12,13^ we performed a randomized clinical trial to test the effect of ENABLE CHF-PC compared with usual care. For our primary end points, we hypothesized that caregivers receiving the intervention would report better quality of life, improved mood (measured by anxiety and depressive symptoms), and decreased burden compared with usual care over 16 weeks. The preplanned secondary end points were caregiver health and positive aspects of caregiving.

## Methods

### Trial Design and Oversight

This was a 2-site, single-blind randomized clinical trial. We followed the Consolidated Standards for Reporting Trials (CONSORT) reporting guideline for trial conduct and reporting.^14^ Adult patients with New York Heart Association (NYHA) class III/IV and/or American Heart Association (AHA)/American College of Cardiology (ACC) stage C/D heart failure and their family caregivers were randomized to either an intervention or usual care group. Patients received a parallel intervention by a separate nurse coach interventionist (results reported separately) (M. A. Bakitas, DNSc, unpublished data, 2020). The study protocol, including the statistical plan, has been published previously^15^ and is included in Supplement 1. The human participants protocol and data safety monitoring plan were approved by the University of Alabama at Birmingham (UAB) and the Birmingham Veteran’s Affairs Medical Center (BVAMC) institutional review boards. Participants provided written informed consent.

### Participants

From October 2015 to August 2018, patient participants were recruited from the outpatient UAB heart failure clinic, the UAB hospital-based transitional care heart failure clinic, and the BVAMC heart failure clinic. Patient eligibility criteria were as follows: English speaking, aged 50 years or older, clinician-determined NYHA class III/IV and/or AHA/ACC stage C/D heart failure, reliable telephone access, and able to complete baseline questionnaires. Patient exclusion criteria included scoring 3 or less on the Callahan cognitive screen,^16^ noncorrected hearing loss, and documented active Axis I diagnosis (eg, schizophrenia, bipolar disorder, active substance abuse). Caregiver eligibility criteria included identification by the patient as “someone who knows you well and is involved in your medical care,” English speaking, aged 18 years or older, reliable telephone access, and able to complete baseline interviews. Family caregivers were required to have a patient enroll in the trial to participate; however, patients could enroll in the trial without a participating caregiver. Caregiver participants received $10 per completion of each questionnaire at baseline and every 8 weeks thereafter for 48 weeks. The target recruitment was 228 caregivers (114 per group). Assuming 20% attrition, an α of .01, and correlation among 3 repeated measurements on the same participants of 0.5, the sample size provided 80% power to detect a standardized intervention effect of Cohen *d* = 0.44.

### Randomization and Blinding

Patients who had an outpatient heart failure clinic appointment in the following 1 to 2 weeks and who potentially met eligibility criteria were identified in the medical record by project coordinators and approached during their outpatient appointment. After signing informed consent and completing baseline measures, patient participants were randomized by the project manager (S.E.) to either the intervention or the usual care group. Caregivers who signed consent and completed baseline measures were assigned to the same group as the patient. Randomization was concealed and performed using a computer-generated randomization scheme in a 1:1 ratio with block size of 2. Randomization was stratified by site (UAB and BVAMC) and race (white and minority). Participants, physicians, and nurse coach interventionists were not blinded. Data collectors who collected self-report questionnaire data, the principal investigator, and co-investigators were blinded. After the analysis was complete, data were unblinded for final interpretation.

### Intervention and Fidelity Monitoring

The details of the ENABLE CHF-PC intervention for family caregivers have been described in detail elsewhere^15^ following guidelines outlined in the Template for Intervention Description and Replication (TIDieR).^17^ This includes formative studies detailing the theoretical basis on Wagner’s Chronic Care Model^18^ and on National Consensus Project Guidelines for Quality Palliative Care^19^ and the development of the format, content, and telehealth delivery approach.^12,13^ In brief, nurse coaches were paired with intervention-group family caregivers and facilitated a series of 4 weekly, one-on-one, 20- to 60-minute phone sessions guided by a *Charting Your Course–Caregiver* guidebook (available from authors on request). Guidebooks were mailed to participants prior to their first session. Participants were encouraged (but not required) to review session material prior to appointments with their nurse coach. Session 1 focused on introducing and defining palliative care, eliciting the caregiver’s illness understanding and the activities they do to support their care recipient, discussing problem solving using the COPE (creativity, optimism, problem solving, expert information) framework,^20^ and outlining the steps of problem solving.^21^ Session 2 reviewed self-care topics, including healthy eating and nutrition, exercise, smoking, relaxation techniques, how to ask for help, and identifying and building a support team. Session 3 addressed partnering in symptom management, including tracking, assessment, and communication; common physical and emotional symptoms in heart failure; and spirituality. Session 4 focused on the role of values and the family member in patient decision-making, advance care planning, and decision aids. Sessions lasted a mean (range) of 44.1 (15-136) minutes. Subsequent to these 4 sessions, nurse coaches followed up monthly until 48 weeks to address any new or ongoing issues. For intervention-group patients who died during the trial period, coaches conducted a bereavement call to offer condolences and discuss grief issues.

Several National Institutes of Health–recommended fidelity strategies were used to ensure validity and reliability of intervention delivery.^22^ Four registered nurse coaches underwent 28 hours of structured orientation and training overseen by the principal investigator (M.A.B.), caregiving expert co-investigator (J.N.D.-O.), and study staff that included self-study, skills practice, and role play. Nurse coaches were guided during sessions by a script to ensure all prescribed topical content was discussed, which was further reinforced by a standardized charting template. All sessions were digitally recorded, and 10% were randomly selected every 4 months for audit by an external consultant using a study team–developed fidelity checklist. There was no protocol nonadherence, defined as 3 consecutive ratings less than 80% over the course of the study. Nurse coaches convened weekly with the principal investigator and caregiving expert co-investigator to review weekly caregiver participant cases to ensure adherence to the intervention protocol. Investigator blinding was maintained during these supervisory meetings by excluding all mention of identifying information and referring to participants by their study ID numbers.

### Primary End Points

Questionnaires were administered by telephone by a blinded data collector at baseline and every 8 weeks thereafter for 48 weeks. Primary end points were quality of life, mood, and burden over 16 weeks. Quality of life was measured using the 15-item Bakas Caregiving Outcomes Scale (BCOS) (scores range from 15-105, with higher scores indicating higher quality of life).^23^ Items assess life changes as a result of providing care to the care recipient (eg, “my self-esteem,” “my future outlook,” “my physical functioning”) on a 7-point scale ranging from “changed for the worst” to “changed for the best.” The BCOS has demonstrated high reliability, with an internal consistency of 0.88. Mood was assessed using the widely used 14-item Hospital Anxiety and Depression Scale (HADS), which has demonstrated strong validity and reliability and uses 7 items to measure anxiety symptoms and 7 items to measure depressive symptoms (subscale scores range from 0 to 21, with scores >8 indicating clinically relevant symptoms).^24^ Caregiver burden was measured using the 14-item Montgomery-Borgatta Caregiving Burden (MBCB) Scale that consists of 3 subscales: objective burden, indicating interference with the caregiver’s day-to-day routine (α = .88; scores range from 6 to 30, with >23 indicating high burden); demand burden, indicating the strain felt by meeting care recipient requests and needs (α = .74; scores range from 4 to 20, with >20 indicating high burden); and stress burden, or emotional stress from caregiving demands (α = .84; scores range from 4 to 20, with >13.5 indicating high burden).^25,26^

### Prespecified Secondary End Points

Prespecified secondary end points included caregiver health using the 10-item PROMIS Global-10 instrument, which measures general domains of health including physical, mental, and social health, symptoms, and overall quality of life. Summed scores are converted to a T-score where 50 is the mean and 10 is the standard deviation (higher scores indicate better health). Positive perceptions of one’s caregiving experience were assessed using the 9-item Positive Aspects of Caregiving Scale (PACS) (scores range from 9 to 45, with higher scores indicating more positive perception of caregiving).^27^

### Statistical Analysis

Descriptive statistics and measures of effect size were used to compare the study groups at baseline. Patterns of missing data due to patient death or attrition were examined. Association between baseline variables and patterns of missing data was assessed with measures of effect size. Longitudinal intention-to-treat analyses were conducted to examine intervention effects using linear mixed-effects modeling for repeated measures at 8 through 16 weeks after randomization with indicators for time, group, and time by group interactions. Pooled standard deviations at baseline were used to compute effect sizes (Cohen *d*) for mean between-group differences in change from baseline to week 8 and 16 combined. Since minimal clinically important differences for the BCOS and HADS have not been established in family caregivers of patients with heart failure, we used a distribution-based approach, set as Cohen *d* = 0.35 (the midpoint between a small effect size [*d* = 0.2] and a medium effect size [*d* = 0.5]).^28^ Per our a priori statistical plan, all testing was 2-sided and conducted at a statistical significance level of .01 without adjustment for multiple inferences.

## Results

### Study Participants

From October 2015 to August 2018, a total of 158 caregivers were randomized, 82 to the intervention group and 76 to the usual care group. (Figure). All were included in the intent-to-treat analysis. Caregiver baseline characteristics are shown in Table 1, with no relevant demographic differences between groups. Family caregivers had a mean (SD) age of 57.9 (11.6) years and were predominantly female (135 [85.4%]), African-American (82 [51.9%]), protestant (145 [91.8%]), and the spouse or partner of the patient (103 [65.2%]). More than half of the total sample provided 5 or more hours of care per day every day of the week. Fifty of the 82 intervention group caregivers (60.1%) completed all 4 intervention sessions.

**Figure.**
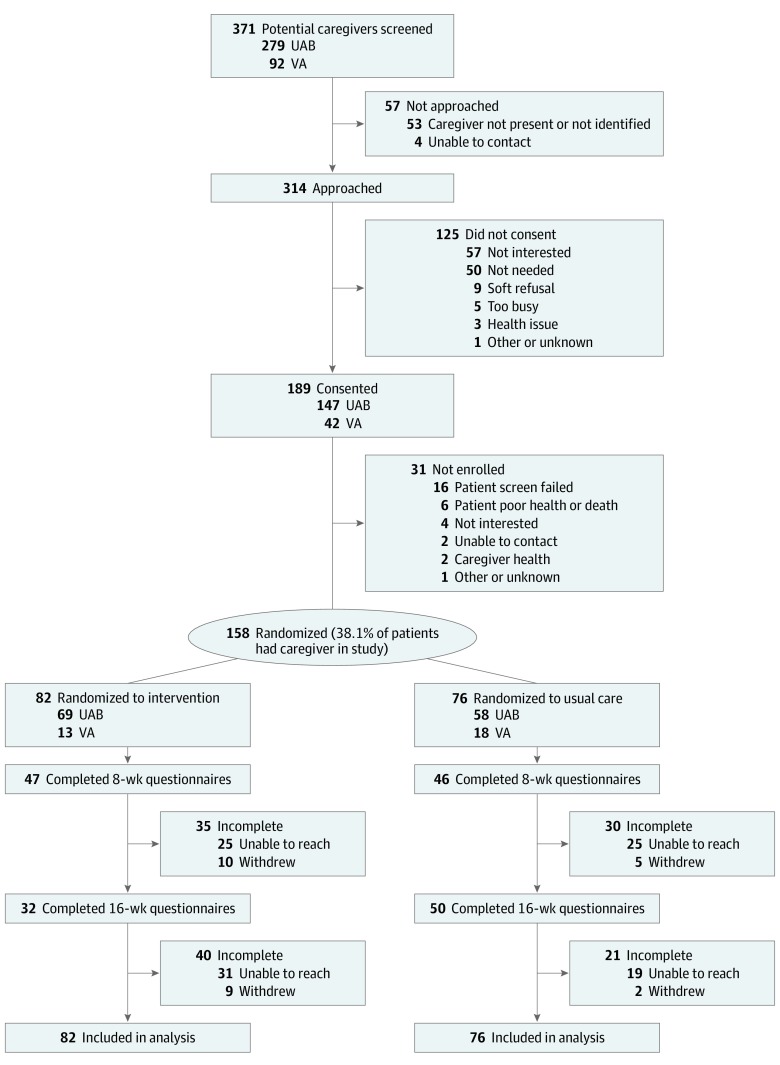
CONSORT Diagram of Study Enrollment Participants were randomized into 2 groups: the intervention group, in which the nurse-led Educate, Nurture, Advise Before Life Ends Comprehensive Heartcare for Patients and Caregivers (ENABLE CHF-PC) intervention was delivered by telephone to the caregiver, and the usual care group, which received standard heart failure care and no other intervention. To maximize potential data points, participants could remain in study after 1 missed data collection window, which contributed to higher 16-week questionnaire completion in participants randomized to usual care. All available data were included in analysis, including data from caregivers whose care recipient died during the study period. UAB indicates University of Alabama, Birmingham, medical center; VA, Veterans Affairs medical center.

**Table 1.** Demographic Characteristics of Caregiver Participants

Characteristic	No. (%)		Effect size, *d*^a^
	Intervention (n = 82)	Usual care (n = 76)	
Age, mean (SD), y	58.2 (12.4)	57.6 (10.8)	0.06
Sex			
Female	73 (89.0)	62 (81.6)	0.21
Male	9 (11.0)	14 (18.4)	
Race			
White	32 (39.0)	38 (50.0)	0.13
African American	46 (56.1)	36 (47.4)	
Other	3 (3.6)	2 (2.6)	
Missing or no response	1 (1.2)	0	
Marital status			
Married or living with partner	57 (69.5)	55 (72.4)	0.11
Never married	9 (11.0)	12 (15.8)	
Divorced or separated	9 (11.0)	6 (7.9)	
Widowed	7 (8.5)	3 (3.9)	
Education			
Less than high school graduate	7 (8.5)	7 (9.2)	0.16
High school or General Education Development	23 (28)	19 (25.0)	
Some college or technical school	40 (48.8)	30 (39.5)	
College graduate or above	12 (14.6)	20 (26.3)	
Employment status			
Full or part time	25 (30.5)	30 (39.5)	0.11
Retired	33 (40.2)	27 (35.5)	
Not employed	5 (6.1)	4 (5.3)	
Disability	19 (23.2)	15 (19.7)	
Religious affiliation			
Protestant	75 (91.5)	70 (92.1)	0.05
Catholic	1 (1.2)	2 (2.6)	
None	2 (2.4)	1 (1.3)	
Other	2 (2.4)	3 (3.9)	
Missing or no response	2 (2.4)	0	
Relationship to patient			
Spouse or partner	53 (64.6)	50 (65.8)	0.16
Parent	16 (19.5)	8 (10.5)	
Other	13 (15.9)	18 (23.6)	
Time spent providing care, d/wk			
≤1	4 (4.9)	7 (9.2)	0.11
2-3	8 (9.8)	10 (13.2)	
4-5	11 (13.4)	5 (6.6)	
6	2 (2.4)	3 (3.9)	
Every day	57 (69.5)	51 (67.1)	
Time spent providing care, h/d			
<1	9 (11.0)	8 (10.5)	0.03
1-2	12 (14.6)	16 (21.1)	
3-4	18 (22.0)	20 (26.3)	
5-6	12 (14.6)	10 (13.2)	
7-8	12 (14.6)	9 (11.8)	
>8	18 (22.0)	12 (15.8)	
Missing or no response	1 (1.2)	1 (1.3)	

^a^ For continuous variables, Cohen *d* is the between-group mean difference divided by the pooled standard deviation; for categorical variables, *d* equivalent is computed as a transformation of a *P* value and sample size.

As shown in Table 2, caregiver baseline measures were also well balanced between groups, except for higher mean (SD) BCOS scores in the intervention group (65.0 [12.9] vs 60.7 [10.4] in the control group; *d* = 0.37). A sensitivity analysis demonstrated that adjusting for baseline BCOS had negligible effects on final results (*d* < 0.03). Overall, the sample at baseline demonstrated low anxiety and depressive symptoms and low burden: at baseline, the mean (SD) HADS-anxiety scores were 3.9 (3.1) in the intervention group and 3.7 (2.9) in the usual care group; HADS-depression scores were 4.7 (3.1) in the intervention group and 4.8 (3.3) in the usual care group; and MBCB mean scores in both groups were below clinical thresholds for high burden. The study collected data beyond the preestablished end point evaluation at 16 weeks. Data collected at weeks 24 and 32 after baseline were used in the modeling to include follow-up information on 7 caregivers who had missing data at both weeks 8 and 16 but provided data at weeks 24 or 32, as well as for 17 participants who had missing data at 16 weeks but provided data at weeks 24 or 32. By week 32, there were 15 caregivers whose patients died (4 [4.8%] in the intervention and 11 [14.4%] in usual care). Among the 143 caregivers whose patients were alive at week 32 (78 in the intervention and 65 in usual care), 54 (37.8%) participated consistently in data collection (ie, provided data on at least 4 of the 5 points; 23 of 78 [29.5%] in the intervention vs 31 of 65 [47.7%] in usual care); however, most (113 [79.1%]) provided data on at least 1 follow-up point (54 of 78 [69.2%] in the intervention vs 59 of 65 [90.8%] in usual care). Conceptually relevant baseline variables associated with consistent participation in data collection were HADS-anxiety (higher scores associated with inconsistent data collection; *d* = 0.4), HADS-depression (higher scores associated with inconsistent data collection; *d* = 0.4), and Positive Aspects of Care (lower scores associated with inconsistent data collection; *d* = 0.4). Because baseline HADS-anxiety and HADS-depression were highly correlated, only HADS-depression and Positive Aspects of Care were used as covariates in the models as appropriate, ie, the models for each of these 2 outcomes used the baseline values of the other as a covariate; for all other outcomes the 2 baseline variables were used as covariates.

**Table 2.** Comparison of Outcomes at Baseline

Measure	Instrument range (clinical cut points)	Score, mean (SD)		Effect size, *d*
		Intervention (n = 82)	Usual care (n = 76)	
Bakas Caregiving Outcomes Scale	15-105 (no cut point)	65.0 (12.9)	60.7 (10.4)	0.37
Hospital Anxiety and Depression Scale				
Anxiety	0-21 (>7 = high)	3.9 (3.1)	3.7 (2.9)	0.07
Depression	0-21 (>7 = high)	4.7 (3.1)	4.8 (3.3)	0.03
Montgomery-Borgatta Caregiver Burden Scale				
Burden type				
Objective	6-30 (>23 = high)	20.1 (2.8)	20 (2.9)	0.04
Demand	4-20 (>15 = high)	11.6 (2.5)	11.6 (1.8)	0
Stress	4-20 (>13.5 = high)	12.3 (2.4)	12.3 (2.2)	0
PROMIS Global Health instrument				
Physical	0-100 (T score; 50 = population mean)	46.9 (8.9)	48 (8.6)	0.13
Mental	0-100 (T score; 50 = population mean)	48.5 (7.1)	48.1 (7.9)	0.05
Positive aspects of caregiving	9-45 (NA)	39 (7.0)	38.5 (6.7)	0.07

Abbreviation: NA, not applicable.

### Study End Points

Table 3 shows model-predicted outcome means for primary and secondary outcomes by time point and group, estimated change from baseline (averaged over weeks 8 and 16) by group, and between-group comparisons of change from baseline to weeks 8 and 16 combined. With regard to the primary outcomes, at week 16, the mean (SE) BCOS score was 66.9 (2.1) in the intervention group and 63.9 (1.7) in the usual care group; over 16 weeks postbaseline, the mean (SE) BCOS score improved 0.7 (1.7) points in the intervention group and 1.1 (1.6) points in the usual care group (difference −0.4; 95% CI, −5.1 to 4.3; SE, 2.4; *d* = −0.03). No relevant between-group differences were observed between the intervention and usual care groups for the HADS-anxiety measure (mean [SE] improvement from baseline, 0.3 [0.3] vs 0.4 [0.3]; difference, −0.1 [0.5]; *d* = −0.02) or depression measure (mean [SE] improvement from baseline, −0.2 [0.4] vs −0.3 [0.3]; difference, 0.1 [0.5]; *d* = 0.03). Results were similar for mean (SE) differences between groups using the MBCB burden scales (objective burden, −0.1 [0.4]; *d* = 0.0; demand burden, 0.2 [0.3]; *d* = −0.18; stress burden, 0.1 [0.3]; *d* = −0.16). With respect to the secondary outcomes, there were small between-group differences in change (*d* range, −0.22 to 0.0) that were unlikely to be clinically relevant. No relevant between-group differences were observed at subsequent points (24 and 32 weeks postbaseline [data not shown]).

**Table 3.** Outcomes From Baseline to 16 Weeks, Intervention vs Usual Care

Outcome	Time after baseline, wk	Intervention			Usual care			Between-group difference in change from baseline^a^		
		Participants, No.	Score, mean (SE)	Change from baseline, mean (SE)	Participants, No.	Score, mean (SE)	Change from baseline, mean (SE)	Mean (SE)	Effect size, *d*	*P* value
Bakas Caregiving Outcomes Scale	0	82	65.2 (1.3)	NA	76	61.2 (1.4)	NA	NA	NA	NA
	8	46	65.3 (1.7)	0.7 (1.7)	46	60.5 (1.7)	1.1 (1.6)	−0.4 (2.4)	−0.03	.88
	16	32	66.9 (2.1)		50	63.9 (1.7)				
Hospital Anxiety and Depression Scale										
Anxiety	0	82	3.9 (0.3)	NA	76	3.7 (0.3)	NA	NA	NA	NA
	8	47	4.5 (0.4)	0.3 (0.3)	46	3.9 (0.4)	0.4 (0.3)	−0.1 (0.5)	−0.02	.88
	16	32	3.8 (0.5)		50	4.2 (0.4)				
Depression	0	82	4.7 (0.3)	NA	76	4.8 (0.4)	NA	NA	NA	NA
	8	47	4.6 (0.4)	−0.2 (0.4)	46	4.5 (0.4)	−0.3 (0.3)	0.1 (0.5)	0.03	.86
	16	32	4.5 (0.5)		50	4.4 (0.4)				
Montgomery-Borgatta Caregiver Burden Scale										
Objective	0	82	20 (0.3)	NA	76	19.9 (0.3)	NA	NA	NA	NA
	8	46	19.8 (0.4)	−0.1 (0.4)	46	19.9 (0.4)	−0.1 (0.4)	0 (0.5)	0	>.99
	16	32	20.2 (0.5)		50	19.7 (0.4)				
Demand	0	82	11.6 (0.2)	NA	76	11.5 (0.3)	NA	NA	NA	NA
	8	46	11.7 (0.3)	−0.1 (0.3)	46	12 (0.3)	0.2 (0.3)	−0.4 (0.4)	−0.18	.35
	16	32	11.1 (0.4)		50	11.6 (0.3)				
Stress	0	82	12.2 (0.3)	NA	76	12.2 (0.3)	NA	NA	NA	NA
	8	46	12.1 (0.3)	−0.3 (0.3)	46	12.5 (0.3)	0.1 (0.3)	−0.4 (0.4)	−0.16	.38
	16	32	11.7 (0.4)		50	12.2 (0.3)				

Abbreviation: NA, not applicable.

^a^ Intervention minus usual care group; change between groups was calculated as mean follow-up (weeks 8 and 16) minus baseline; *P* values are from the time by group interaction term in longitudinal models; *d* was calculated as mean difference in change from baseline divided by baseline pooled standard deviation.

## Discussion

In an effort to address national priorities to develop models of support for family caregivers of individuals with serious illness,^29^ we conducted the largest, most racially diverse randomized clinical trial to date of an intervention to support family caregivers of community-dwelling persons with advanced heart failure.^10,11^ The results showed that a nurse-led, early palliative care telehealth intervention (ENABLE CHF-PC) did not demonstrate significant differences in quality of life, mood, and burden compared with usual care over 16 weeks. Prespecified secondary outcomes including global health and positive aspects of caregiving were also not significantly different at 16 weeks. While the intervention did not demonstrate benefit to these particular outcomes over a 16 week time frame, the results nonetheless reveal essential insights to advancing future intervention testing and to guiding clinical services for heart failure family caregivers.

A plausible explanation for the lack of intervention effect was that our cohort of family caregivers was not distressed nor experiencing poor quality of life. Hence, our intervention had no room to improve the distress and quality of life of these individuals, resulting in a possible floor effect. Furthermore, slightly more than half of the sample was African American, a population that has been observed in prior studies to have lower levels of distress and higher quality of life compared with white populations (despite reporting higher levels of caregiving intensity).^30,31^ As observed in Table 2, the mean scores for anxiety, depression, and objective, demand, and stress burden were all below the cut points for clinical importance. Only 21 of the 158 caregiver participants (13.3%) had depressive symptom scores greater than the clinical threshold at baseline. These low rates of distress in our sample indicate that our cohort was significantly better off than what has been previously observed in this population.^4,32^ Furthermore, participants in the parallel patient intervention reported higher baseline levels of perceived quality of life and mood compared with other palliative care intervention trials^33,34^ (Kansas City Cardiomyopathy Questionnaire: mean [SD], 52.6 [21]; HADS-depression: mean [SD], 5.7 [4.3]) (Marie A. Bakitas, DNSc, unpublished data, November 11, 2019), suggesting these patients may not have needed as much intensive assistance from caregivers. A critical implication is that future intervention and clinical services should screen for those caregivers with significant distress who may benefit more from structured support.

Another possible reason the intervention did not demonstrate benefit over usual care was that only a small proportion of intervention group caregivers completed the entirety of the 4 core intervention sessions. Only 50 (60.1%) intervention group caregivers completed all 4 intervention sessions. This raises the possibility that an inadequate dose of the intervention was delivered over the 16-week time frame.^35^ The intervention was developed as a multicomponent intervention with each session delivering specific content. Because nearly 40% of caregivers received a smaller dose of the intervention than intended, the intervention’s potency may have been too attenuated to signal change in the sample. Furthermore, there may be particular sessions and topical content that are more effective in triggering benefit than others that many caregivers did not receive. It is also possible that the intervention itself was too burdensome, which may partially explain the higher attrition in this group, particularly for those who had more distress. Going forward, it will be imperative to understand the individual contributions of intervention components and their interactions in future palliative care interventions, potentially using optimization trial designs that are configured to test individual component effects and screen out overly burdensome components.^36^ Such designs in future research would allow for more robust examination of palliative care intervention dose-effect relationships and effective components.

Interventions to support caregivers of persons with heart failure have largely been unsuccessful and yielded inconsistent findings.^2,10,11^ However, our results yield insights to future intervention testing and to the development of clinical services for this population. First, it is likely that not all caregivers need robust clinician support; therefore, caregivers who are most likely to benefit from an intervention should be identified and targeted. Caregivers who may benefit more from support are those who surpass thresholds for clinical high distress; who endorse a number of unmet instrumental, knowledge, decisional readiness, or skill training needs; or who are faced with assisting with critical decisions (eg, left ventricular assist device placement) or repeated seminal events (eg, care recipient hospitalizations).^37,38^ Second, to increase adherence, interventions and clinical services need to be designed in a way that adapts to caregivers’ limited amount of time and flexibly delivered around a caregivers’ schedule. Third, the impact of caregiving interventions may be more noticeable downstream as patients become sicker near the end of life and the demands and strains of caregiving become more acute. Fourth, caregiving interventions might also be dually designed to optimize both the caregiver’s well-being and health and also their performance as a deliverer of high-quality care to patients. Mounting research suggests that heart failure family caregivers play a critical role in promoting patient outcomes such as medication and treatment adherence, health, survival, self-management behaviors, and quality of care.^2,9,11,39^ Thus, interventions designed for caregivers should target these critical patient outcomes.

### Limitations

Our study has several limitations. First, our sample of caregivers was limited to 2 sites and 1 region of the country and may not generalize elsewhere. Second, our sample of 158 caregivers was less than our a priori targeted sample of 228. This increases the statistical uncertainty of our results; therefore, our findings do not completely exclude the possibility of a meaningful effect of early palliative care. Third, there may be selection bias in caregivers who were willing to participate. While our analysis of demographic variables associated with participation did not yield any differences, there may be unmeasured variables associated with participation. Fourth, there were 6 more participants in the intervention group than in the control group. Caregivers were assigned to the group that patients were randomized to after patients completed their baseline measures. Intervention group patients’ interactions with nurse coaches may have indirectly influenced more intervention group caregivers to ultimately participate. This may have resulted in a mild imbalance in baseline BCOS scores, which a sensitivity analysis showed had negligible impact on final results. All other characteristics and outcomes appeared balanced at baseline. In addition, we observed significant missing data from approximately half of participants and found that higher anxiety and depressive symptom scores were associated with higher rates of missing data. While this was adjusted for in the analyses, there may be a selection bias.

## Conclusions

To date, few interventions for heart failure family caregivers have undergone high-quality testing and demonstrated efficacy. To our knowledge, this study was the largest and most racially inclusive trial to date of a nurse coach–led palliative care intervention for family caregivers of patients with advanced heart failure. The study was rigorously conducted but did not show beneficial effects to caregivers over 16 weeks. Insights from our results suggest that future interventions should be briefer, target more distressed caregivers than those in our sample, and assess effects on patient outcomes. Development of reliably efficacious interventions for diverse heart failure family caregivers remains elusive but is of such importance that continued efforts and testing are needed to optimize culturally appropriate support to this hidden workforce.

## Notes

Supplement 1. **Trial Protocol**

Supplement 2. **Data Sharing Statement**
