# Supplementary material for: Effects of a Telehealth Early Palliative Care Intervention for Family Caregivers of Persons With Advanced Heart Failure: The ENABLE CHF-PC Randomized Clinical Trial
Source: JAMA Netw Open. 2020 Apr 13;3(4):e202583. doi: 10.1001/jamanetworkopen.2020.2583 (PMC7154802; doi:10.1001/jamanetworkopen.2020.2583)
Supplement: Supplement 1. — Trial Protocol [file jamanetwopen-3-e202583-s001.pdf]

Additional ENABLE CHF-PC Documentation

Initial IRB Approval & PT/CG Consent Forms 10/1/2014 (pp. 2-15)
Specific Aims (16)
Research Strategy (17-38)

OMB No. 0990-0263
Approved for use through 3/31/2015

Protection of Human Subjects
Assurance Identification/IRB Certification/Declaration of Exemption
(Common Rule)

Policy: Research activities involving human subjects may not be conducted or supported by the Departments and Agencies adopting the Common Rule (56FR28003, June 18, 1991) unless the activities are exempt from or approved in accordance with the Common Rule. See section 101(b) of the Common Rule for exemptions. Institutions submitting applications or proposals for support must submit certification of appropriate Institutional Review Board (IRB) review and approval to the Department or Agency in accordance with the Common Rule.

Institutions must have an assurance of compliance that applies to the research to be conducted and should submit certification of IRB review and approval with each application or proposal unless otherwise advised by the Department or Agency.

1. Request Type	2. Type of Mechanism	3. Name of Federal Department or Agency and, if known, Application or Proposal Identification No.
[X] ORIGINAL<br>[ ] CONTINUATION<br>[ ] EXEMPTION	[X] GRANT [ ] CONTRACT [ ] FELLOWSHIP<br>[ ] COOPERATIVE AGREEMENT<br>[ ] OTHER:____________________	National Institutes of Health

4. Title of Application or Activity	5. Name of Principal Investigator, Program Director, Fellow, or Other
Randomized Trial of ENABLE CHF-PC for Heart Failure Patients and Caregivers	BAKITAS, MARIE A

6. Assurance Status of this Project *(Respond to one of the following)*

[X] This Assurance, on file with Department of Health and Human Services, covers this activity:
Assurance Identification No. FWA00005960 , the expiration date 01/24/2017 IRB Registration No. IRB00000726

[ ] This Assurance, on file with *(agency/dept)*____________________, covers this activity.
Assurance No.__________, the expiration date__________ IRB Registration/Identification No.__________*(if applicable)*

[ ] No assurance has been filed for this institution. This institution declares that it will provide an Assurance and Certification of IRB review and approval upon request.

[ ] Exemption Status: Human subjects are involved, but this activity qualifies for exemption under Section 101(b), paragraph__________.

7. Certification of IRB Review (Respond to one of the following IF you have an Assurance on file)

[X] This activity has been reviewed and approved by the IRB in accordance with the Common Rule and any other governing regulations.
by: [ ] Full IRB Review on (date of IRB meeting) __________ or [X] Expedited Review on (date) 10/1/14
[ ] If less than one year approval, provide expiration date __________

[ ] This activity contains multiple projects, some of which have not been reviewed. The IRB has granted approval on condition that all projects covered by the Common Rule will be reviewed and approved before they are initiated and that appropriate further certification will be submitted.

8. Comments
Protocol subject to Annual continuing review.

Title X140813007
Randomized Trial of ENABLE CHF-PC for Heart Failure Patients and Caregivers

Partial HIPAA Waiver Approved?: Yes

IRB Approval Issued: 10-1-14

IRB Approval No Longer Valid On: 10-1-15

9. The official signing below certifies that the information provided above is correct and that, as required, future reviews will be performed until study closure and certification will be provided.	10. Name and Address of Institution
11. Phone No. *(with area code)* (205) 934-3789<br>12. Fax No. *(with area code)* (205) 934-1301<br>13. Email: irb@uab.edu	University of Alabama at Birmingham<br>701 20th Street South<br>Birmingham, AL 35294
14. Name of Official Julius Linn	15. Title IRB Member
16. Signature [signature]	17. Date 10/1/14

Authorized for local Reproduction Sponsored by HHS

According to the Paperwork Reduction Act of 1995, no persons are required to respond to a collection of information unless it displays a valid OMB control number. The valid OMB control number for this information collection is 0990-0263. The time required to complete this information collection is estimated to average 30 minutes per response. If you have comments concerning the accuracy of the time estimate(s) or suggestions for improving this form, please write to: U.S. Department of Health & Human Services, OS/OCIO/PRA, 200 Independence Ave., S.W., Suite 336-E, Washington D.C. 20201, Attention: PRA Reports Clearance Officer.

PI: BAKITAS, MARIE A
Protocol # X140813007

UAB IRB Approval of Partial Waiver of HIPAA Authorization to Use PHI in Screening for Research

☒ **Patient Authorization: Approval of Partial HIPAA Waiver to Use PHI in Screening for Research.** The IRB reviewed the proposed research and granted the request for a "partial HIPAA waiver," to allow the proposed use of protected health information (PHI) in screening for research, based on the following findings:

1. The use/disclosure of PHI to screen candidates for research involves no more than minimal risk to the privacy of individuals
   a. There is an adequate plan to protect the identifiers from improper use and disclosure.
   b. There is an adequate plan to destroy the identifiers at the earliest opportunity consistent with conduct of the research, unless there is a health or research justification for retaining the identifiers or such retention is otherwise required by law.
   c. The PHI will not be reused or disclosed to any other person or entity, except as required by law, for authorized oversight of the research study, or for other research for which the use or disclosure of PHI would be permitted.
2. The screening cannot practicably be conducted without the waiver or alteration.
3. The screening cannot practicably be conducted without access to and use of the PHI.

—OR—

☐ **Full Review**

The IRB reviewed the proposed research at a **convened meeting** at which a majority of the IRB was present, including one member who is not affiliated with any entity conducting or sponsoring the research, and not related to any person who is affiliated with any of such entities. The partial waiver of authorization for screening was approved by the majority of the IRB members present at the meeting.

____
Date of Meeting

____
Signature of Chair, Vice-Chair or Designee

____
Date

☒ **Expedited Review**

The IRB used an **expedited review procedure** because the research involves no more than minimal risk to the privacy of the individuals who are the subject of the PHI for which use or disclosure is being sought. The review and approval of the partial waiver of authorization for screening was carried out by the Chair of the IRB, or by one of the Vice-Chairs of the IRB as designated by the Chair of the IRB.

10/1/14
Date of Expedited Review

[signature]
Signature of Chair, Vice-Chair or Designee

10/1/14
Date

PATIENT CONSENT FORM

**TITLE OF RESEARCH:**	Randomized Trial of ENABLE CHF-PC for Heart Failure Patients and Caregivers.
**IRB PROTOCOL NO.:**	X140813007
**INVESTIGATOR:**	Marie Bakitas, DNSc, CRNP
**SPONSOR:**	National Institutes of Health/National Institute of Nursing Research

Purpose of the Research

You are being asked to take part in a **research study. Taking part in research is voluntary**.

You are being asked to take part in this study because you have been diagnosed with a condition known as heart failure. Your decision whether or not to take part will have no effect on the quality of your medical care. Please ask questions if there is anything about this study you do not understand. You may discontinue your participation in the study in general or in any portion of the intervention phone calls or questionnaires at any time.

The **purpose of this study** is to learn how to improve supportive care for patients and caregivers as they live with heart failure. We have developed a phone-based, educational program to help patients and their caregivers have a better quality of life and mood and lower symptom burden, in addition to the care services that you currently receive at UAB. The goal of this study is to determine if this additional education program improves patient and caregiver outcomes. Nearly 1000 patients with cancer and their caregivers have experienced this type of care; however, this approach is relatively new for patients with heart failure.

We expect 190 patients with 190 caregivers at UAB to enroll. Half of the participants in this study will have access to these additional services and half of participants in this study will have access to supportive services that are currently available at UAB.

Explanation of Procedures

Your participation in this study may last up to 48 weeks. If you decide to enroll into this research study, you will be assigned by chance (like the flip of a coin) by a computer to one of two groups (either Group A or Group B). Neither you nor your doctor can control into which group you will be assigned.

UAB IRB
Date of Approval 10-1-14
Not Valid On 10-1-15

Group A – receives the normal standard of heart failure and supportive care that you would receive if you did not enroll in this study, including access to palliative care, social work, pastoral, and financial planning services.

Also you will be asked to complete questionnaires (at the beginning and at weeks 8, 16, 24, 32, 40, and 48) about your symptoms, quality of life and mood, and quality of your medical care.

Group B – receives additional supportive services, which includes two components: One In-person comprehensive Palliative Care Team (PCT) Consultation by a palliative care team member with special training in supportive care, as soon as feasible after enrollment, performed at UAB and telephone sessions with a Nurse Coach (NC) who will cover materials found in a guidebook called *Charting Your Course* (CYC) that you will receive. These calls will be scheduled at a day/time that is convenient for you. Also you will be asked to complete questionnaires (at the beginning and at weeks 8, 16, 24, 32, 40, and 48) about your symptoms, quality of life and mood, and the quality of your medical care.

Timeline:

**When**	**Type of meeting**	**Description/Topics**	**Group A**	**Group B**	**Approx. Duration**
Today	In-person	1) Sign consent<br>2) Eligibility screening<br>3) First set of quality of life questionnaires	X	X	1 hr.
Ongoing	Ongoing	Normal standard of heart failure management support	X	X	
As soon as feasible	In-person	In-clinic palliative care team assessment		X	1 hr.
Week 1	Phone	*Charting Your Course* (CYC) Session 1: Problem-solving		X	45 min.
Week 2	Phone	*CYC 2:* Self-care management		X	45 min.
Week 3	Phone	*CYC* 3 Assessing, Prioritizing, and Managing Symptoms		X	45 min.
Week 4	Phone	*CYC 4:* Communication skills, decision-making, and advanced care planning		X	45 min.
Week 5	Phone	*CYC 5:* Discussion and supportive counseling		X	30 min.
Week 6	Phone	*CYC 6:* Discussion and supportive counseling		X	30 min.
Once per month	Phone	Your nurse coach will check-in with you monthly to follow-up		X	15 min.
Weeks 8 to 48	Phone	Quality of life questionnaires	X	X	25-45 min.

Risks and Discomforts

You may become distressed, short of breath, or fatigued when reflecting on your symptoms, mood, and quality of life during the questionnaires or the educational sessions with the nurse coach. If you become distressed you can discuss this with your nurse coach or we can refer you to a counselor. You are also free to stop or pause any session at any time and reschedule should you not feel well or become fatigued during a call. In our experience of providing supportive care interventions, the risks of these occurrences are quite low.

There is a chance that people not associated with the study will see your answers to questionnaires and your medical record information. Your name and other identifying information will be removed from study documents. Data will be kept in locked files in the study research offices at UAB. Participant data will be housed at a data repository at UAB.

There is a risk related to being placed into a group by chance (randomization, like the flip of a coin). Participants placed in the intervention group may receive benefits associated with the extra support activity. The normal standard of heart failure care group may not have the same benefits as the intervention group. Participants in the intervention group may be more burdened by having to participate in extra intervention activities.

Benefits

You may not personally benefit from being in this research study. We hope to gather information that may help us to provide better care to people in the future.

Alternatives

If you decide not to enroll in this study, you will receive the normal standard of supportive heart failure care that is usually provided at UAB.

Confidentiality

Information obtained about you for this study will be kept confidential to the extent allowed by law. However, research information that identifies you may be shared with the UAB Institutional Review Board (IRB) and others who are responsible for ensuring compliance with laws and regulations related to research, including people on behalf of the National Institutes for Health, National Institute of Nursing Research Staff, and the Office for Human Research Protections (OHRP). The results of the study may be published for scientific purposes. However, your identity will not be given out.
Information obtained during the course of the study which, in the opinion of the investigator(s), suggests that you may be at significant risk of harm to yourself or others will be reportable to a third party in the interest of protecting the rights and welfare of those at potential risk.

Voluntary Participation and Withdrawal

Whether or not you take part in this study is your choice. There will be no penalty if you decide not to be in the study. If you decide not to be in the study, you will not lose any benefits you are otherwise owed. You are free to withdraw from this research study at any time. Your choice to leave the study will not affect your relationship with this institution.

You may skip any questions in the questionnaire sessions you do not wish to answer. You are also free to stop or pause any session at any time and reschedule should you not feel well or become fatigued during a call. Calls are scheduled with you ahead of time for times between 8AM and 5PM, Monday through Friday or when convenient for you.

You may be removed from the study without your consent if the sponsor ends the study, if the study doctor decides it is not in the best interest of your health, or if you are not following the study rules.

If you are a UAB student or employee, taking part in this research is not a part of your UAB class work or duties. You can refuse to enroll, or withdraw after enrolling at any time before the study is over, with no effect on your class standing, grades, or job at UAB. You will not be offered or receive any special consideration if you take part in this research.

Cost of Participation

There no fees charged to you for taking part in this study.

Participants using a cell phone for calls related to this study will be responsible for their own cell phone plan charges. The table with the Timeline on page 2 lists the estimated amount of time for phone calls in this study.

The costs of your standard medical care will be billed to you and/or your insurance company in the usual manner.

Payment for Participation in Research

You will be provided with $10 for each of 7 data collection interviews (at baseline, weeks 8, 16, 24, 32, 40, and week 48). Separate $10 check payments will be mailed to you after completion of each data collection interview. If you complete the entire study, you will receive a total of $70.

Significant New Findings

You will be told by your doctor or the study staff if new information becomes available that might affect your choice to stay in the study.

Questions

If you have any questions, concerns, or complaints about the research including available treatments, you may contact Dr. Marie Bakitas. She will be glad to answer any of your questions. Dr. Bakitas' number is 205-934-5277. Dr. Bakitas may also be reached after hours by calling 603-398-7766.

If you have questions about your rights as a research participant, or concerns or complaints about the research, you may contact the UAB Office of the IRB (OIRB) at (205) 934-3789 or toll free at 1-855-860-3789. Regular hours for the OIRB are 8:00 a.m. to 5:00 p.m. CT, Monday through Friday. You may also call this number in the event the research staff cannot be reached or you wish to talk to someone else.

Legal Rights

You are not waiving any of your legal rights by signing this informed consent document.

Signatures

Your signature below indicates you that you have read (or been read) the information provided above and agree to participate in this study. You will receive a copy of this signed consent form.

____

Signature of Participant Date

____

Signature of person obtaining consent Date

____

Signature of Witness Date

Reviewed by:

____

Signature of Principal Investigator Reviewing Consent Document Date

University of Alabama at Birmingham
AUTHORIZATION FOR USE/DISCLOSURE OF HEALTH INFORMATION FOR RESEARCH

What is the purpose of this form? You are being asked to sign this form so that UAB may use and release your health information for research. Participation in research is voluntary. If you choose to participate in the research, you must sign this form so that your health information may be used for the research.

Participant Name: ____________________

Research Protocol: Randomized Trial of ENABLE CHF-PC for Heart Failure Patients and Caregivers.

UAB IRB Protocol Number: X140813007

Principal Investigator: Marie Bakitas, DNSc, CRNP

Sponsor: NIH/National Institute of Nursing Research

What health information do the researchers want to use? All medical information and personal identifiers, including past, present, and future history, examinations, laboratory results, imaging studies and reports and treatments of whatever kind related to or collected for use in the research protocol.

Why do the researchers want my health information? The researchers want to use your health information as part of the research protocol listed above and described to you in the Informed Consent document.

Who will disclose, use and/or receive my health information? The physicians, nurses and staff working on the research protocol (whether at UAB or elsewhere); other operating units of UAB, HSF, UAB Highlands, Children's of Alabama, Eye Foundation Hospital and the Jefferson County Department of Public Health, as necessary for their operations; the IRB and its staff; the sponsor of the research and its employees; and outside regulatory agencies, such as the Food and Drug Administration.

How will my health information be protected once it is given to others? Your health information that is given to the study sponsor will remain private to the extent possible, even though the study sponsor is not required to follow the federal privacy laws. However, once your information is given to other organizations that are not required to follow federal privacy laws, we cannot assure that the information will remain protected.

How long will this Authorization last? Your authorization for the uses and disclosures described in this Authorization does not have an expiration date.

Can I cancel the Authorization? You may cancel this Authorization at any time by notifying the Principal Investigator, in writing, referencing the Research Protocol and IRB Protocol Number. If you cancel this Authorization, the study doctor and staff will not use any new health information for research. However, researchers may continue to use the health information that was provided before you cancelled your authorization.

Can I see my health information? You have a right to request to see your health information. However, to ensure the scientific integrity of the research, you will not be able to review the research information until after the research protocol has been completed.

Signature of participant: ____ Date: __________

or participant's legally authorized representative: ______________________ Date: __________

Printed Name of participant's representative: ______________________

Relationship to the participant: ______________________________

CAREGIVER CONSENT FORM

TITLE OF RESEARCH: Randomized Trial of ENABLE CHF-PC for Heart Failure Patients and Caregivers.

IRB PROTOCOL NO.: X140813007

INVESTIGATOR: Marie Bakitas, DNSc, CRNP

SPONSOR: National Institutes of Health/National Institute of Nursing Research

Purpose of the Research

You are being asked to take part in a **research study. Taking part in research is voluntary.**

You are being asked to take part in this study because you provide care for someone who has been diagnosed with a condition known as heart failure. Please ask questions if there is anything about this study you do not understand.

The **purpose of this study** is to learn how to improve supportive care for patients and caregivers as they live with heart failure. We have developed a phone-based, educational program to help patients and their caregivers have a better quality of life and mood and lower symptom burden, in addition to the care services that you currently receive at UAB. The goal of this study is to determine if this additional education program improves patient and caregiver outcomes. Nearly 1000 patients with cancer and their caregivers have experienced this type of care; however, this approach is relatively new for patients with heart failure.

We expect 190 patients with 190 caregivers at UAB to enroll. Half of the participants in this study will have access to these additional services and half of participants in this study will have access to supportive services that are currently available at UAB.

Explanation of Procedures

Your participation in this study may last up to 48 weeks. If you decide to enroll into this research study, the patient will be assigned by chance (like the flip of a coin) by a computer to one of two groups (either Group A or Group B). You will be part of the group to which the patient was assigned. Neither you nor the patients' doctor can control into which group you will be assigned.

UAB IRB
Date of Approval 10-1-14
Not Valid On 10-1-15

Group A – receives the normal standard of heart failure and supportive care that would be provided to a caregiver if you did not enroll in this study, including access to palliative care, social work, pastoral, and financial planning services.

Also you will be asked to complete questionnaires (at the beginning and at weeks 8, 16, 24, 32, 40, and 48) about your quality of life and mood.

Group B – receives additional supportive services, which includes two components: One In-person comprehensive Palliative Care Team (PCT) Consultation by a palliative care team member with special training in supportive care, as soon as feasible after enrollment, performed at UAB and telephone sessions with a Nurse Coach (NC) who will cover materials found in a guidebook called *Charting Your Course* (CYC) that you will receive. These calls will be scheduled at a day/time that is convenient for you. Also you will be asked to complete questionnaires (at the beginning and at weeks 8, 16, 24, 32, 40, and 48) about your quality of life and mood.

Timeline:

**When**	**Type of meeting**	**Description/Topics**	**Group A**	**Group B**	**Approx. Duration**
Today	In-person	1) Sign consent<br>2) Eligibility screening<br>3) First set of quality of life questionnaires	X	X	1 hr.
Ongoing	Ongoing	Normal standard of heart failure management support	X	X	
As soon as feasible	In-person	In-clinic palliative care team assessment (patient only unless they wish for you to be present)		X	1 hr.
Week 1	Phone	*Charting Your Course* (CYC)Session 1: Caregiver role and problem-solving		X	45 min.
Week 2	Phone	*CYC 2:* Caregiver self care		X	45 min.
Week 3	Phone	*CYC 3*: Being a partner in symptom management		X	45 min.
Week 4	Phone	*CYC 4:* Communication, support, and decision-making		X	45 min.
Once per month	Phone	Your nurse coach will check-in with you monthly to follow-up		X	15 min.
Weeks 8 to 48	Phone	Quality of life questionnaires	X	X	25-45 min.

Risks and Discomforts

You may become sad or upset after reflecting on the care you provide the person with heart failure during the questionnaires or the educational sessions with the nurse coach. In our experience of providing supportive care interventions, this risk is quite low. If you become distressed you can discuss this with your nurse coach or we can refer you to a counselor.

There is a chance that people not associated with the study will see your answers to questionnaires. Your name and other identifying information will be removed from study documents. Data will be kept in locked files in the study research offices at UAB. Participant data will be housed at a data repository at UAB.

There is a risk related to being placed into a group by chance (randomization, like the flip of a coin). Participants placed in the intervention group may receive benefits associated with the extra support activity. The normal standard of heart failure care group may not have the same benefits as the intervention group. Participants in the intervention group may be more burdened by having to participate in extra intervention activities.

Benefits

You may not personally benefit from being in this research study. We hope to gather information that may help us to provide better care to people in the future.

Alternatives

If you decide not to enroll in this study, you and the individual with heart failure will receive the normal standard of heart failure supportive care that is usually provided at UAB.

Confidentiality

Information obtained about you for this study will be kept confidential to the extent allowed by law. However, research information that identifies you may be shared with the UAB Institutional Review Board (IRB) and others who are responsible for ensuring compliance with laws and regulations related to research, including people on behalf of the National Institutes for Health, National Institute of Nursing Research Staff, and the Office for Human Research Protections (OHRP). The results of the study may be published for scientific purposes. However, your identity will not be given out.

Information obtained during the course of the study which, in the opinion of the investigator(s), suggests that you may be at significant risk of harm to yourself or others will be reportable to a third party in the interest of protecting the rights and welfare of those at potential risk.

Voluntary Participation and Withdrawal

Whether or not you take part in this study is your choice. There will be no penalty if you decide not to be in the study. If you decide not to be in the study, you will not lose any benefits you are otherwise owed. You are free to withdraw from this research study at any time. Your choice to leave the study will not affect your relationship with this institution.

You may skip any questions in the questionnaire sessions you do not wish to answer. You are also free to stop or pause any session at any time and reschedule should you not feel well or become fatigued during a call. Calls are scheduled with you ahead of time for times between 8AM and 5PM, Monday through Friday or when convenient for you.

You may be removed from the study without your consent if the sponsor ends the study, if the study doctor decides it is not in the best interest of your health, or if you are not following the study rules.

If you are a UAB student or employee, taking part in this research is not a part of your UAB class work or duties. You can refuse to enroll, or withdraw after enrolling at any time before the study is over, with no effect on your class standing, grades, or job at UAB. You will not be offered or receive any special consideration if you take part in this research.

Cost of Participation

There no fees charged to you for taking part in this study.

Participants using a cell phone for calls related to this study will be responsible for their own cell phone plan charges. The table with the Timeline on page 2 lists the estimated amount of time for phone calls in this study.

Payment for Participation in Research

You will be provided with $10 for each of 7 data collection interviews (at baseline, weeks 8, 16, 24, 32, 40, and week 48). Separate $10 check payments will be mailed to you after completion of each data collection interview. If you complete the entire study, you will receive a total of $70.

Significant New Findings

You will be told by your doctor or the study staff if new information becomes available that might affect your choice to stay in the study.

Questions

If you have any questions, concerns, or complaints about the research including available treatments, you may contact Dr. Marie Bakitas. She will be glad to answer any of your questions. Dr. Bakitas' number is 205-934-5277. Dr. Bakitas may also be reached after hours by calling 603-398-7766.

If you have questions about your rights as a research participant, or concerns or complaints about the research, you may contact the UAB Office of the IRB (OIRB) at (205) 934-3789 or toll free at 1-855-860-3789. Regular hours for the OIRB are 8:00 a.m. to 5:00 p.m. CT, Monday through Friday. You may also call this number in the event the research staff cannot be reached or you wish to talk to someone else.

Legal Rights

You are not waiving any of your legal rights by signing this informed consent document.

Signatures

Your signature below indicates you that you have read (or been read) the information provided above and agree to participate in this study. You will receive a copy of this signed consent form.

____

Signature of Participant Date

____

Signature of person obtaining consent Date

____

Signature of Witness Date

Reviewed by:

____

Signature of Principal Investigator Reviewing Consent Document Date

University of Alabama at Birmingham
AUTHORIZATION FOR USE/DISCLOSURE OF HEALTH INFORMATION FOR RESEARCH

What is the purpose of this form? You are being asked to sign this form so that UAB may use and release your health information for research. Participation in research is voluntary. If you choose to participate in the research, you must sign this form so that your health information may be used for the research.

Participant Name: ____________________

Research Protocol: Randomized Trial of ENABLE CHF-PC for Heart Failure Patients and Caregivers.

UAB IRB Protocol Number: X140813007

Principal Investigator: Marie Bakitas, DNSc, CRNP

Sponsor: NIH/National Institute of Nursing Research

What health information do the researchers want to use? All medical information and personal identifiers, including past, present, and future history, examinations, laboratory results, imaging studies and reports and treatments of whatever kind related to or collected for use in the research protocol.

Why do the researchers want my health information? The researchers want to use your health information as part of the research protocol listed above and described to you in the Informed Consent document.

Who will disclose, use and/or receive my health information? The physicians, nurses and staff working on the research protocol (whether at UAB or elsewhere); other operating units of UAB, HSF, UAB Highlands, Children's of Alabama, Eye Foundation Hospital and the Jefferson County Department of Public Health, as necessary for their operations; the IRB and its staff; the sponsor of the research and its employees; and outside regulatory agencies, such as the Food and Drug Administration.

How will my health information be protected once it is given to others? Your health information that is given to the study sponsor will remain private to the extent possible, even though the study sponsor is not required to follow the federal privacy laws. However, once your information is given to other organizations that are not required to follow federal privacy laws, we cannot assure that the information will remain protected.

How long will this Authorization last? Your authorization for the uses and disclosures described in this Authorization does not have an expiration date.

Can I cancel the Authorization? You may cancel this Authorization at any time by notifying the Principal Investigator, in writing, referencing the Research Protocol and IRB Protocol Number. If you cancel this Authorization, the study doctor and staff will not use any new health information for research. However, researchers may continue to use the health information that was provided before you cancelled your authorization.

Can I see my health information? You have a right to request to see your health information. However, to ensure the scientific integrity of the research, you will not be able to review the research information until after the research protocol has been completed.

Signature of participant: ______________________________ Date: __________

or participant's legally authorized representative: ______________________ Date: __________

Printed Name of participant's representative: ____________________

Relationship to the participant: ______________________________

SPECIFIC AIMS

Significance: More than 5 million Americans, most over age 65, carry a diagnosis of heart failure (HF).[1] Despite treatment advances, up to 40% of patients will die within 1 year of first hospitalization.[2] Age[3], race,[4] and rural location[5] are risk factors associated with the greatest HF morbidity and mortality.[6-8] HF is the most common reason for hospital admissions in persons over age 65[9] resulting in unrelieved physical and emotional suffering[4,10] and immense social and economic costs.[11-17] However, palliative care (PC) been shown to alleviate many of the symptoms that reduce quality of life (QOL) in advanced HF,[18-21] including elements of recommended HF care management[22] that are often lacking, such as self-care activation, decision support, communication, advanced care planning and care coordination.[23-30] Despite this, there has been little progress in incorporating these essential palliative care services into HF care, especially early in the disease trajectory when there is the greatest potential for benefit.[16,29,31-33] Only 19% of Medicare-aged HF patients (and their family caregivers) access hospice, compared with more than half of advanced cancer patients.[34] This gap is a critical issue and hence we are proposing this high impact pragmatic randomized controlled trial (RCT), responsive to NINR's mission…"to stimulate research on optimal palliative and end of life care models based on measurable outcomes".

We[35] and others[36,37] have demonstrated that early concurrent palliative cancer care achieves beneficial outcomes in QOL, symptom burden, depression, and in some cases survival. Based on Wagner's Chronic Illness Care (CIC) Model,[35] ENABLE (Educate, Nurture, Advise, Before Life Ends)[38-40]--a nurse-led, multicomponent, telehealth care model, recognized *in a recent consensus statement*[41] *and Cochrane Systematic Review*[42]-- provides foundational evidence for the World Health Organization (WHO) continuum of care model.[43] The WHO model posited that introducing key palliative care concepts early in the disease trajectory would increase opportunities for patients and families to benefit from all that palliative care has to offer. ENABLE is not prognosis-dependent or focused on end-of-life (EOL).[44] Rather it provides one on one coaching with patients and their family caregivers, targeting knowledge and skills in symptom management, self-care, decision-making and problem solving, communication, advance care planning, and care coordination in order to live as well as possible with chronic illness. This non-threatening approach can obviate the barriers to palliative care integration in HF. *A completed pilot study of ENABLE: CHF-PC (**C**omprehensive **H**eartcare **F**or **P**atients and **C**aregivers) allowed us to revise and test all of the proposed study procedures and instruments in advanced HF patients who were early in the advanced HF trajectory and their caregivers, thus demonstrating successful adaptation of this model to a predominantly underserved HF population.*[44]

Innovation: We expect that this innovative pragmatic RCT will address critical knowledge gaps in implementing effective early, concurrent palliative care in an underserved advanced HF population in a number of ways. First, we will strategically embed the intervention within two health care delivery systems: an academic, tertiary referral center (University of Alabama at Birmingham (UAB)) and the Birmingham Veteran's Affairs Medical Center (BVAMC), both of which provide care to large populations of rural, minority HF patients. Second, we specifically designed the model to address caregiver needs, characteristics, and key outcomes (e.g. QOL, mood, burden, *self-reported health)*. Third, we will explore mediating and moderating factors (e.g. *health literacy,*[45] social support,[46] coping style[22], activation,[18] etc.) known to specifically influence HF intervention outcomes. And fourth, we will continue to explore and develop novel statistical methods that jointly consider QOL and survival.[47] Specific aims, relative to HF care as usual are:

Specific Aim 1 Determine whether ENABLE: CHF-PC leads to higher advanced HF **patient-reported** QOL and mood (depression/anxiety); and lower symptom burden and resource use (e.g. hospital admissions and days, emergency visits) at 8 and 16 weeks after baseline. ***-Hypothesis 1*** *Intervention participants will experience higher QOL and mood, and lower symptom burden and resource use at 8 and 16 weeks after baseline compared with those receiving usual HF care.*

Specific Aim 2 Determine whether ENABLE: CHF-PC leads to higher **caregiver-reported** QOL, mood (anxiety/depression), and *self-reported health* and lower caregiver burden at 8 and 16 weeks after baseline. -***Hypothesis 2*** *Intervention caregivers will report higher QOL, mood, and self-reported health, and lower caregiver burden at 8 and 16 weeks after baseline.*

Exploratory Aims:

EA 1 Explore mediators and moderators of patient and caregiver outcomes and reciprocal relationships.
EA 2 Examine intervention effects using joint modeling approaches.

Investigative team: We will leverage the expertise of our established palliative care, HF, and geriatric investigative teams and consultants with vast experience in conducting pioneering multi-center palliative care intervention trials.[32,35-3748-53] We are well-positioned to build on a solid foundation of palliative care science, using the strategy of a pragmatic trial, to improve QOL, reduce symptom burden, and optimize resource use in *a real world setting* of underserved older adults and their family caregivers.

SIGNIFICANCE

A1. HF in older (>65 years) adults is an understudied public health crisis.

Over 5 million Americans, most over age 65, carry a diagnosis of heart failure (HF);[1] up to 40% of patients will die within 1 year of first hospitalization.[2] Age[3], race[4] and rural environment[5] are risk factors associated with the greatest HF morbidity and mortality.[6-8] In persons over age 65;[9] multiple hospitalizations often result in unrelieved physical and emotional suffering[4,10] and immense social and economic costs.[11-17] However little is known about age-dependent HF outcomes or treatment response because clinical exclusion criteria favor younger patients with less co-morbidity.[18-20]

A2. Palliative care is highly likely to improve symptom burden, anxiety, depression, and QOL in older adults.

Despite strong evidence for palliative treatments such as opioids for pain and dyspnea; psychotherapy, antidepressants, and multi-component, counseling interventions,[21] few are part of routine HF care.[22,23] Symptoms, especially dyspnea, increase with progressive HF,[24-26] and may equal or exceed those reported by cancer patients.[12,16,27-29] Dyspnea was identified as the source of 'unbearable suffering' in 52% of Dutch HF patients requesting assisted suicide.[30] Pain,[13,31] anxiety,[32-34] and depression[11,33,35] are also under-recognized and under-treated in HF leading to adverse effects on QOL, reduced adherence to medical regimens, and lower survival. In one large study,[31] 50% of veterans with HF reported moderate to severe pain (≥4/10); pain was more frequently reported than dyspnea. There is growing evidence that providing palliative care early and concurrent with routine HF care, especially in older adults, can improve HF symptoms and QOL,[22,27,36,37] relieve physical and emotional suffering,[11-17] and possibly reduce hospitalizations.[10] However there are currently few care models that consistently provide this care.

A3. ENABLE CHF-PC provides 2013 ACCF/AHA Guideline[38]-recommended HF palliative care

Proponents of concurrent palliative HF care;[15,22,28,39-44] and the *2013 ACCF/AHA guidelines specifically identify that palliative care for HF should include attention to "…symptom assessment, psychosocial distress, HRQOL, preferences about end-of-life care, caregiver support, and assurance of access to evidence-based disease-modifying interventions" (p.e207).* The myriad challenges to providing this care are: 1) the episodic, unpredictable HF illness trajectory[45] in patients who are not "labeled" as dying[43,46] (and their professional and informal caregivers); 2) overcoming the barriers of late referral due to prognostic uncertainty[47,48] or until all HF therapies have been exhausted;[23] and 3) a lack of systematic, prospective patient engagement in shared decision-making and advance care planning.[48] ENABLE CHF-PC is an evidence-based model that meets or exceeds the *2013 ACCF/AHA guidelines for palliative HF care and overcomes these challenges.*[49-51]

A4. ENABLE CHF-PC, based on the CIC, normalizes early introduction of vital palliative care principles.

In HF, adequately-powered intervention studies and meta-analyses examining Wagner's Chronic Illness Care (CIC) model have demonstrated improved *disease-focused* HF self-management and survival outcomes; [52-58] some gains have equaled guideline-based drug therapy.[58,59] To our knowledge, ENABLE was the first early concurrent palliative care intervention to successfully apply the CIC model to an advanced oncology[60-62] and now an advanced HF population.[63] ENABLE-CHF-PC is a multi-component, coaching approach comprising an in-person palliative care team (PCT) assessment[64] and phone-based, patient and caregiver sessions (described in Approach). These components are complementary and reinforcing; the in-person PCT consultation provides a guideline-based[65] comprehensive assessment and builds a foundation for future consultation if needed and the phone-based coaching sessions provide comprehensive information in an unhurried, convenient home setting. These components are standardized but tailored to individual patient and caregiver needs (see *Charting Your Course* patient and caregiver guidebooks and coaching manuals in Appendix B). Similar to oncologists[66] and advanced cancer patients,[67] *clinicians, patients and caregivers in our recently completed HF pilot study*[68] described ENABLE as non-threatening and empowering. ENABLE-CHF PC maintains the original CIC framework fostering patient activation and empowerment,[62] effective communication about advanced care planning, treatment goals, prognosis, and symptom management.[69-71] However, HF clinicians are reluctant to discuss these topics and their struggle to identify the "right time" often leaves HF patients and their families unaware of potentially beneficial supportive palliative care services.[37,71] Our approach normalizes the early introduction of 2013 ACCF/AHA Guideline-recommended palliative care content[38],[32,33,37] (e.g. dyspnea management, informed decision-making, etc.) by embedding palliative nurse coaches within the cardiology team. Thus HF clinicians and patients can be exposed to palliative care principles early but still have the "*assurance of access to evidence-based disease-modifying interventions".*[38]

A5. ENABLE targets disparities created by rural location and low health literacy**.** The Deep South, and Alabama in particular, has the lowest prevalence of palliative care in large (50%) and second lowest in small (4%) hospitals in the United States.[72] Among the myriad, complex issues contributing to Alabama's "D" grade (A (best) to F (failing) scale),[72] indicative of poor overall palliative care performance, rural location[73-75] and low health literacy are two major factors that greatly contribute to this public health crisis. Rural Healthy People 2010 identified HF as a leading healthcare priority[75] for the 40% of the population who live in rural areas.[76] Rural advanced HF patients are at high risk

for social isolation and disparities in palliative care[77,5] due to long distances, low population density,[78] and few local palliative care specialists or primary-care or hospice clinicians with palliative care expertise.[73-75],[5] Rural location is associated with less and later hospice use[79] and hospitalized death.[80] The challenge of rural location is often compounded by patients having low health literacy. Low literacy in HF patients has been estimated to be between 34-54%.[81] This is particularly concerning due to mounting evidence linking HF patients' low health literacy with higher hospitalization rates[82-84] and health care costs,[81] poor self-care and disease management skills,[84-86] worse quality of life, [86] and higher mortality.[82,87] Recognizing this, the Heart Failure Society of America (HFSA)[88] issued a consensus statement recommending the development of interventions that evaluate social support, patient follow-up, disease management, and educational tools—all of which are included in the proposed ENABLE CHF-PC intervention. Specifically, components of ENABLE CHF-PC have demonstrated success in combating the effects of rural location[50,64,73] and low literacy: self-care training,[89,90] multi-session training,[89,90] and phone-based support.[90]

<u>A6 There is a critical need to discover effective interventions to assist caregivers of patients with advanced HF</u>
The bedrock of successful HF self-care is having a family caregiver;[53,91] but caregivers' invaluable contributions to patients' well-being come at a personal cost. [92,93] High levels of caregiver strain have been associated with poorer physical health[94] and higher mortality risk.[95,96] A number of interventions aimed at supporting family caregivers of patients with life limiting illnesses have undergone randomized testing, however the results have been marginal.[21,97] A 2011 Cochrane review and meta-analysis[97] of 11 RCTs comparing interventions for family caregivers of terminally ill patients with usual care (n=1836) showed significant though small improvements in psychological distress (standardized mean difference, -0.15) and non-significant small improvements in QOL (-0.11). One possible explanation for these small intervention effects may relate to the late timing of these interventions relative to the patient's advanced illness trajectory. Thus early intervention, such as we propose, has been suggested as a means to reduce or prevent the caregiver strain and distress that has been noted to peak at the diagnosis of advanced illness and as death approaches. [98-101] Intervention timing is equally important as content. To date HF-specific caregiver interventions have primarily focused on improving patients' disease-related self-management outcomes (e.g. sodium self-management)[102] rather than specifically targeting *caregiver* burden, QOL, and bereavement. One exception is the successful problem-solving approach[103-105] that was originally applied in hospice family caregivers[106,107] and which we adapted, tested and will use in this proposal of caregivers early in the HF disease trajectory.[68] While our pilot demonstrated a positive impact on HF patients' caregiver mood, burden and QOL outcomes in the setting of introducing palliative care content much earlier than has been previously offered, caregivers continued to report that this content would have been appreciated even earlier in the diagnosis (see Preliminary Work).

<u>A7 There is surprisingly little data-based theory on the operative mechanisms of palliative care</u>**.** The AHA Statement on *"Promoting Self-Care in Persons with Heart Failure"* identified the importance of determining the operative mechanisms of effective tele-health and self-management strategies.[108-110] While ENABLE improved QOL, mood, and in some cases survival,[63] the operative mechanisms of these effects had not yet been fully explored. Our proposal directly addresses this issue. As depicted in Figure 1a, we hypothesize, based on the CIC model[111,112] that the intervention may have relatively immediate effects on patients' behaviors (activation, goal setting, problem solving, follow-up /coordination, and decision support) and adoption of these behaviors may operate as proximal mediators and have more distal effects on patients' sense of social support and coping. Ultimately, these proximal and distal intervention effects are hypothesized to serve as the mechanisms whereby the intervention improves patients' QOL and reduces depression, anxiety, and perceived symptom burden. Evidence for these mechanisms has been found in studies of patients in rural primary care,[113] HF[55] and others.[114,115] Resultant improved communication and advance care planning has also been associated with improved QOL, lower use of aggressive treatments at EOL, and increased hospice length of stay.[116-122] Increased social support[123-125] and coping style[126-128] have been associated with improved cardiac patients' well-being. To our knowledge no studies have prospectively addressed caregiver intervention mechanisms. Figure 1b illustrates hypothesized caregiver, patient, and reciprocal interactions.[129] Examining these relationships may potentially identify mutable factors for future caregiver intervention targets. [130,131]

Figure 1a Specific Aim 1 & Exploratory Aim Patient Conceptual Model

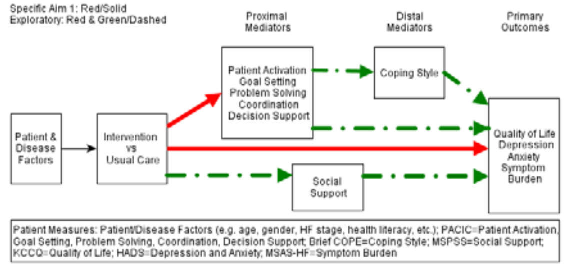

Figure 1b Specific Aim 2 Caregiver Conceptual Model

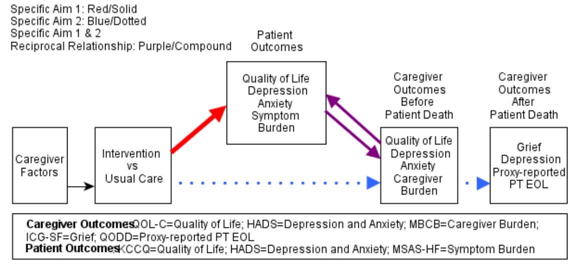

A8 This proposal addresses the knowledge gap related to longitudinal analysis of palliative care data.
Miaskowski[132,133] and others at the NINR-sponsored summit, *"Science of Compassion: Future Directions in End-of-Life and Palliative Care"* made a plea for the use of increasingly sophisticated statistical analysis methods for longitudinal palliative care patient data. Our group has implemented innovative analysis methods in our previous work[63] and we will continue to develop these methods in the HF population (See Tosteson and Li consultant LOS/biosketches).

B INNOVATION

B1 Few concurrent palliative care interventions, using a pragmatic design, have been tested in underserved Deep South HF populations. Our investigative team (including consultant Dr. Christine Ritchie[134-138]-see LOS/biosketch) is unique as one of the few in the country with the necessary depth, scope, leadership, and experience to conduct a high impact pragmatic RCT of early integration of palliative care in underserved advanced-illness patients.[68,73,139-141] As described in our Preliminary Work, we have a long history of conducting successful multi-site palliative care trials that reach rural, underserved patients. Whether or not our trial demonstrates positive outcomes, adopting a more pragmatic design by embedding palliative nurse coaches within the clinical cardiology departments and identifying data collection opportunities within the electronic health record will provide important insights about resource use, sustainability and future model dissemination / implementation.[142,143]

B2 Explicating palliative care mechanisms, especially in HF caregivers, is novel and necessary in developing effective palliative care models. The exact mechanisms whereby ENABLE improved QOL, depression, and survival[50,63,64] remains to be explored. Based on recent data,[38,87,144] we have included *health literacy,* in addition to social support, and coping as theorized mutable intervention "active ingredients". ENABLE specifically targets these factors which may in turn mediate patient and caregiver outcomes *(including the often unmeasured caregiver-reported health).* Understanding these mechanisms is essential to creating maximally efficient, effective palliative care models.

B3 Developing innovative statistical approaches for longitudinal data analysis of palliative care outcomes
A systematic review of 22 palliative care RCTs[145] identified significant methodological shortcomings in longitudinal data analysis. Analyses of the dependence between quality and quantity of life are typically neglected in palliative care clinical trials but as we have proposed[146] must be interpreted jointly to most accurately evaluate the primary scientific hypotheses. Specific properties of the joint distribution of the patient-reported QOL and survival data are: QOL conditional on survival, QOL in the months before death, overall survival and combined measures of survival and QOL such as quality-adjusted life years (when utilities are measured). Further, we propose to continue developing this innovative statistical methodology using "terminal decline" models[146,147] that incorporate the unique HF pattern of decline which may ultimately suggest causal pathways thus improving our understanding of intervention effect.

B4 Summary of Significance and Innovation
This innovative RCT addresses the urgent need to integrate palliative care into the routine care of underserved, older adults with HF in the following ways: First, ENABLE: CHF-PC will be the first pragmatic RCT to study an extensively tested, evidence-based, tele-health palliative care coaching intervention, tailored to older underserved adults, that introduces novel problem-solving, decision support techniques/patient decision aids, and life review early in the advanced HF trajectory as recommended by the 2013 ACCF/AHA Guideline[38]. Second, ENABLE CHF-PC has a specific caregiver component, with a special focus on caregiver *health literacy and self-reported health*. Third, this proposal contributes to palliative care science by examining factors that may impact patient and caregiver intervention mechanisms (e.g. *health literacy*, coping, social support) and novel analytic methods that can inform future intervention development and testing. Finally, this multi-site study brings together an established inter-professional investigative team representing palliative care, HF, geriatrics, and behavioral psychology within an enthusiastic, outstanding research intensive infrastructure to address the special needs of underserved older adults with HF and their family caregivers. Therefore, as we have already demonstrated in palliative and EOL, multi-site, VA, rural, community-based and cancer studies this proposal can have a high impact by shifting the paradigm of HF care.

C APPROACH

PRELIMINARY WORK relevant to the proposed RCT.

C1.1 ENABLE Trials in cancer. We have systematically developed the evidenced-based ENABLE palliative care intervention through two large clinical trials[49-51,63] and a recently completed, multi-site RCT (R01NR011871-01) (See figure below). The ENABLE I RWJ-funded demonstration project (1999-2001)[49,51] provided the primary feasibility data that led to the ENABLE II efficacy RCT (NCI R01 CA101704, Ahles/Hegel (PI); Bakitas-(co-I)) published in the *Journal of the American Medical Association*[63], ASCO Consensus Statement,[74] Cochrane Review,[148] and is now under review for the NCI Research-tested Intervention Program (RTIPs). Thus, this model has become a standard of high quality cancer care.[36,149] We have just completed the ENABLE III RCT investigating the effect of immediate vs delayed introduction of palliative care. **ENABLE III**

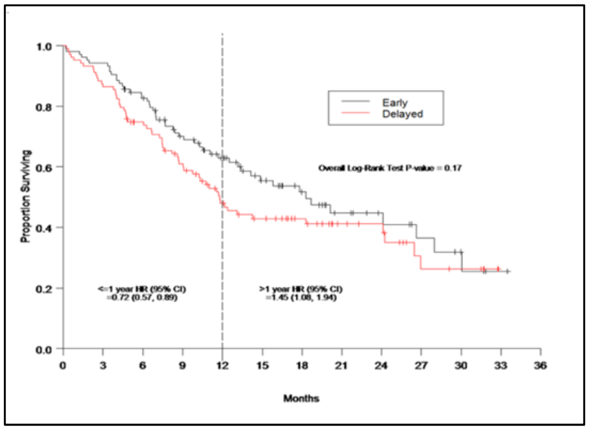

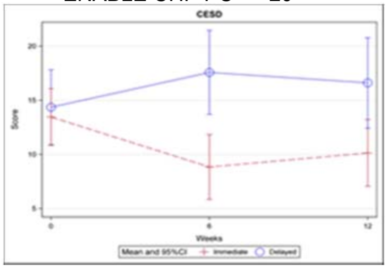

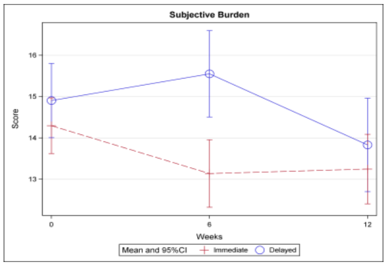

Preliminary Results: demonstrated a statistically significant survival advantage at 1 year for immediate entry patients (Figure). Compared to delayed entry patients, the relative risk of death (hazard ratio [HR] (95% CI)) was lower for immediate participants at 1 year 0.72 (95% CI, 0.57-0.89) (*P*=0.003) despite small to moderate intervention effects at 12 weeks ((mean [SD]: .13 (21.39) for QOL (*P*=.34), -.21 (3.63) for symptom impact (*P*=.09), and .04 (3.91) for depressed mood (*P*=.33).[150] Importantly, as shown in the Figures, there were statistically significant treatment effects of immediate entry from randomization to 12 weeks on caregiver depression: -4.1 [1.3] for depression (CESD) (*P*=.003), -1.0 [0.4] for subjective burden (SB) (*P*=.02), and a trend in QOL.[151] **Relevance to the current study:** Provides a rigorous foundation for the intervention and methods [36,51,63,149,152-157] to be used in the proposed trial.

C1.2 ENABLE CHF- PC: Needs assessment: Prior to our original submission we conducted a needs assessment to investigate HF patients (ICD-9 codes 428.0 – 428.99) potential palliative care needs and utilization.[158] **Results:** Between 2006-2010 of 1763 HF admissions representing 1320 unique patients 132 had 516 palliative care consultation visits (approximately 27 unique patient consults/year). Nearly half of the patients were male (n=70), married (n=70) with a mean age of 75 years at the time of consultation. Most consultations occurred during a hospital admission and were for a symptom management crisis, goals of care discussion, or to assist with transition to home hospice care. **Relevance to the current study:** Provides observational data validating that palliative care is routinely introduced late in the HF trajectory.

C1.3 ENABLE CHF-PC Formative Evaluation Pilot. We completed a 2-phase formative evaluation pilot study[159] in which we revised and tested ENABLE CHF-PC, study procedures and instruments in advanced HF patients and a family caregiver. In Phase I we tailored the study materials for a HF population based on expert consultation and literature review, and clinician feedback (N=11). In Phase II we evaluated the intervention, study procedures and instruments in two consecutive waves of 5 patient/caregiver dyads. **Results:** Phase I clinician interviews revealed the many challenges of providing PC to patients with HF such as knowing the right time and approach to address palliative care issues such as advance care planning given the unpredictable trajectory of their illness, the limited palliative services in rural areas, and the stigma of the "palliative care" label. Phase II: HF patients and caregivers expressed having a positive experience with the phone-based intervention but wished it had been available earlier in the illness trajectory. Small to moderate intervention effect sizes were noted (see Table). **Relevance to the current study:** Provides essential evidence demonstrating feasibility and acceptability of the intervention, recruitment/retention strategies, and study procedures. Identifies signals for small-moderate improvements in relevant outcomes.

Patient and Caregiver Outcome Measures

Group	Measure	Baseline Mean Score (SD) (*N*=11)	Mean Standardized Effect Size (*d*) from Baseline	
			Week 12 (*N*=6)	Week 24 (*N*=5)
HF partients	HADS Total	25.11 (4.38)	0.54	0.01
	Anxiety	12.67 (3.66)	0.36	-0.16
	Depression	12.44 (1.67)	0.66	0.41
	KCCQ Summary Score	51.94 (18.95)	0.31	0.06
	MSAS Summary Score	2.04 (0.46)	-0.22	0.33
	PACIC Summary Score	3.49 (0.53)	0.47	-0.08
Family caregivers	HADS Total	24.89 (7.2)	0.05	0.12
	Anxiety	10.67 (3.68)	-0.06	0.23
	Depression	14.22 (3.81)	0.14	-0.06
	MBCB Total	45.78 (6.3)	0.25	0.28
	Objective Burden	17.67 (5.27)	0.04	0.41
	Subjective Burden	15.89 (2.58)	0.01	-0.96
	Demand Burden	12.22 (1.52)	0.64	0.96
	QOLC Total	82.06 (22.2)	-0.06	0.33

C1.4 The Alabama HF Project (Ahmed): **C1.4 The Alabama HF Project (Ahmed):** As a part of his NHLBI-funded R01, Dr. Ahmed analyzed data from the Alabama HF Project that is based on 8049 Medicare beneficiaries discharged alive with a primary discharge HF diagnosis in 1998-2001 from 106 Alabama hospitals.[177] We conducted a post hoc analysis of these data to examine the role of palliative care on hospital readmissions for hospitalized HF patients. We compared HF patients who received a discharge hospice referral with a propensity-matched cohort of HF patients who would have been hospice-eligible (that is, those who died within six months post-discharge). We noted hospice referral was associated with 88% lower risk of 30-day all-cause readmissions when compared with hospice-eligible patients who died within 6 months post-discharge and did not receive a hospice referral. In that study, about a quarter of the patients died within 6

months of hospital discharge and yet only 8% of those patients (2% of all patients) received hospice referral.[178]
<u>Relevance to the current study</u>: Provides evidence of potential for palliative care services to reduce 30-day readmissions; created an opportunity for the PI to establish new collaborations with UAB/BVAMC investigators.

<u>C1.5 Social Support, PAtients' Needs, and Hospital Use for Heart Failure (SSPAN-HF) (AHA) and the DCH Social Support Study (NIA R03AG031995)</u> (Durant) Based on prior work, [160-165] two on-going survey studies are examining the relationships among social support, HF self-care, and hospital use among racially-diverse HF patients at UAB Hospital and a rural referral center. In both projects, Dr. Durant cumulatively enrolled 586 African American (AA) and white HF patients. Of that total cohort, 210 AAs were recruited from UAB and 128 AA from the rural referral site over a 12-month period. UAB Hospital data from June 2012 to June 2013 reveals that 1009 unique AAs (most over age 65), were admitted for HF at least once during this 12-month period. To explore the quantitative survey findings about HF knowledge, self-efficacy and self-care, 40 semi-structured qualitative interviews with a subset of AA men with HF, we noted the variation and inadequacy of HF information provided by health care professionals ("nobody ever explained"), difficulties distinguishing HF symptoms from those likely caused by co-morbidities (e.g. "chest pain", "heart fluttering") and insufficient self-care measures in response to symptoms (e.g. "relax", "take a nitroglycerin","call 911").
<u>Relevance to the current study:</u> Demonstrates availability and ability to recruit adequate numbers of minority HF patients from the proposed sites and provides a HF cohort of 700 patients, mostly AAs, who have agreed to be contacted for future HF studies and will be available to be contacted for the proposed study.

<u>C1.6 Multisite BVAMC palliative care intervention studies</u> Drs. Bailey and Burgio have conducted a series of VA-funded palliative care research studies for the last 10 years. *BEACON* (Best Practices for End-of-Life Care for our Nation's Veterans), was a 7-site intervention trial to evaluate a multi-component education-based intervention to improve inpatient EOL care processes.[166] Hundreds of hospital providers were trained to identify actively dying patients and to implement best practices from home-based hospice care, supported by an electronic order set and paper-based educational tools. Based on chart abstraction of 6,067 deceased veterans (20% with HF), the intervention resulted in significant improvements in several processes of care.[140] The next phase of this research (including Drs. Bakitas/Kvale-co-Is; proposal under review, HSRD) is a national, 48-VAMC site implementation project. The team has also pursued several studies of a hospice emergency kit (HEK) developed by Dr. Bailey and implemented as a BVAMC standard of care for veterans being discharged to home hospice. The most recent published study was a prospective cohort of 45 patient/caregiver dyads (12% with HF) in home hospice (enrolled in less than 9 months)[166]provided promising evidence that HEKs are a feasible, well-tolerated method for achieving timely relief of emergent symptoms and possibly avoiding unwanted ED visits and hospitalizations. Finally, the BVAMC participated in a multi-site RCT of discontinuation of statins in home hospice patients conducted by the NINR-funded *Palliative Care Research Consortium* (Kvale/Goode-site-PIs). The BVAMC was one of the top recruiters, demonstrating our ability to screen and enroll eligible palliative care patients for clinical trials.
Relevance to the proposed study: Demonstrates successful collaboration among the proposed study investigators and expertise in conducting multi-site palliative care intervention studies within and outside the VA system.

Summary of Preliminary Work: ENABLE, one of the first community-based concurrent palliative care models to demonstrate effectiveness in reducing the burden of serious illness for patients and caregivers, has undergone more than a decade of systematic intervention development and effectiveness research in cancer and formative evaluation in advanced HF. The UAB/BVAMC teams have conducted important HF/palliative care studies using population-based methods, demonstrated the ability to recruit and deliver interventions to minority HF populations, and to conduct multi-site palliative care research. We are poised to undertake a full scale pragmatic RCT in underserved HF patients and caregivers.

<u>C2 STUDY DESIGN</u>

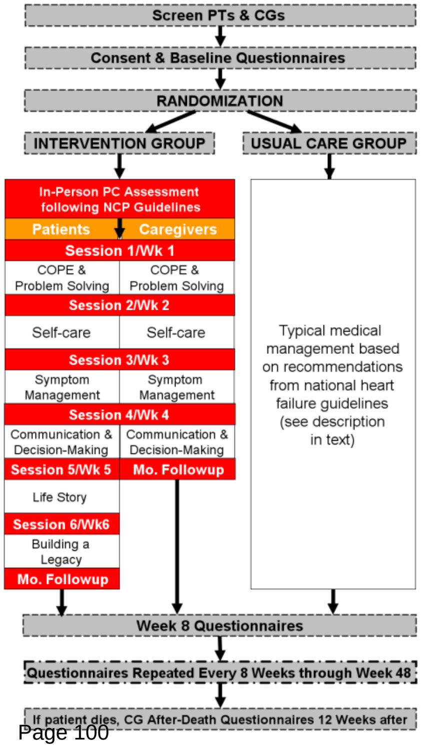

The figure illustrates the study schema for this prospective pragmatic RCT of ENABLE CHF-PC, a theoretically-based early palliative care model that is integrated with advanced HF care compared to usual care. Our goal is to determine intervention effects on patient QOL, symptom burden, anxiety, depression and resource use (primary) outcomes and caregiver QOL,

burden, anxiety/depression, and self-reported health. Based on our prior work[51,63] the CIC model[111,112], *and recently completed formative evaluation pilot study[68]* ENABLE CHF-PC comprises: 1) an in-person **P**alliative **C**are **T**eam **(PCT)** assessment and 2) an Advanced Practice **P**alliative Care **N**urse **C**oach **(PNC)** embedded in the UAB and BVAMC HF clinical teams instituting a phone-based, 6-session patient curriculum and 4-session caregiver curriculum; both followed by monthly follow up calls that will reinforce prior content. ENABLE CHF-PC is not focused on terminal illness; but rather it addresses patient and family-centered care issues of living well with chronic, progressive illness, empowerment, problem-solving, symptom management and self-care, defining personal goals, and decision-making.

C2.1 Justification of a pragmatic RCT design

We[63] and others[148] have carried out rigorous early palliative care trials using an experimental approach yielding statistically significant results. However the true test of benefit is to determine if these care models are able to be applied and show benefit in real-life clinical practice.[142] To our knowledge we will be among the first to use a pragmatic design in an early palliative care HF RCT. As we illustrate in Appendix A, the proposed trial maintains some elements of an explanatory trial (e.g. structured intervention, PNC specialized training, fidelity monitoring), as defined by the 10 domains of the PRECIS (pragmatic-explanatory continuum indicator summary).[143] However other elements will maintain characteristics typical of real-world current U.S. HF care (e.g. no attempt to control HF practitioner care, participant compliance, or primary outcomes (intent to treat) analysis). We hope that the knowledge to be gained by this design will contribute to the current sparse data on palliative care integration into HF and accelerate the practical applicability of palliative care in routine practice.

C2.2 Specific Aim 1 Patient Sample and Justification

Patients ≥65 years will be recruited from the UAB and BVAMC cardiology, advanced HF, geriatric, and primary care clinics and from Dr. Durant's (co-I)[160,163]existing UAB HF study cohorts. HF occurs most commonly in older adults and the Seattle Heart Failure model[167] has demonstrated some ability to identify patients who might benefit from palliative care.[41] These broad criteria, consistent with a pragmatic design, balance the competing issues of sample homogeneity and external validity/generalizability.

Patient Inclusion Criteria: 1) English-speaking; 2) able to complete baseline interview (see Instruments-Appendix E); 3) Age ≥65; 4) NYHF III/IV or ACC Class D HF (physician-determined); 5) Seattle Heart Failure Model ≥ 50% 2 year mortality (http://depts.washington.edu/shfm/)[41,167]

Patient Exclusion Criteria: 1) Dementia or significant confusion (measured by ≤3 Callahan score [168] 2) DSM-IV Axis I diagnosis (e.g. schizophrenia, bipolar disorder, or active substance use disorder); 3) Patients will not be excluded if they do not identify a caregiver.*

* We have consciously chosen to make allowances (in this and our prior intervention studies) in favor of a pragmatic approach recognizing that many chronically-ill patients have little or no caregiver support. We recognize that caregiver support can positively influence some HF outcomes and if imbalance.[39,110,169] .

Specific Aim 2 Caregiver Sample and Justification

Consistent with our prior studies[50,51,63 102] and a pragmatic design we will use a broad definition of "caregiver".

Caregiver Inclusion Criteria: 1) Identified by the patient as "a person who knows you well & is involved in your medical care". May live in the same household and/or be considered to be primary caregiver willing to participate;"[102] 2) English-speaking; 3) able to complete baseline interview (see Instruments-Appendix E)

C2.3 Total Enrollment

We anticipate a total enrollment of 380 patient participants and 228 caregivers over 42 months (from our previous studies, ~60% caregivers participated). Approximately half of the patient participants (n=190) will be randomized to the intervention and half (n=190) will be randomized to receive usual HF care. Likewise, due to stratified randomization by indication of caregiver, approximately half of caregivers (n=114) will be randomly assigned to intervention and the other half to usual care (control) (n=114).

Justification of Enrollment Estimates

We have carefully examined our three sources of recruitment for volumes and racial diversity to assure that we can meet our enrollment of 380. This sample size will also allow us to estimate and test intervention effects and to conduct subgroup analyses (e.g. by race) with sufficient power to detect meaningful differences.

UAB: Primary HF diagnosis (age ≥65) is among the top five UAB hospital discharges accounting for 943 patients per year of which 38% are African American (AA). The UAB HF program follows over 1000 ambulatory HF patients. Conservatively these overlapping cohorts comprise an estimated 1400 patients per year; approximately 80% (n=1120) will meet eligibility criteria (stage III/IV or Class D). Assuming a steady rate of presentation of 93 patients per month over 42 months of recruitment =3920 total eligible UAB HF patients.

BVAMC: Of 300 active HF patients approximately 80% will meet eligibility criteria or 240 total HF patients over 42 months. Existing UAB HF Cohort: Co-investigator Dr. Raegan Durant has an established cohort from prior studies of approximately 700 HF patients (40% AA) that are willing to be contacted for future studies. Hence, approximately 4920 HF patients will be eligible over 42 months from the combined sources. We have consistently enrolled just under 50% (in this case 2460) of eligible patients over the last 10 years of palliative care trials. [50,51,63] Conservatively, if only 30% of the 2460 eligible patients consent, that would provide a possible pool of 738 patients to achieve the enrollment target of 380.
Attrition In prior palliative care studies we have experienced 10% attrition due to withdrawal or loss to follow up over 2 years.[50,51,63] We will oversample if needed to achieve a final sample of 380 to evaluate primary patient outcomes and vital status.

C2.3 Recruitment and retention

Prior successful methods will be employed to maximize recruitment and retention and minimize missing data. UAB and VA clinicians are co-I's and cardiology leadership is enthusiastic and supportive of this study which is the key to patient access and successful recruitment (See LOS). Recruitment will be facilitated by experienced research coordinators on-site and/or available by pager during clinic hours. The research coordinators will screen provider schedules to identify patients meeting eligibility criteria. Flyers and Clinician Information Sheets describing eligibility and study information will be posted in all exam and work rooms, and waiting areas. Eligible patients in the existing HF cohort will be contacted via letter or phone to determine interest. To enhance retention, research coordinators will be maintaining contact via data-collection, monitoring patients' status through the electronic medical record, and their clinician.

C3 Intervention Protocol and Procedures

ENABLE CHF PC includes two major components:
1) In-person comprehensive Palliative Care Team (**PCT**) Consultation- as soon as feasible after enrollment; performed at the UAB or BVAMC Supportive Care Clinics (See Facilities and Resources).

2) A **P**alliative Care **N**urse **C**oach (**PNC**) embedded within the advanced UAB and BVAMC HF teams, instituting a phone-based 6-session patient curriculum and a 4-session caregiver curriculum followed by monthly phone-based supportive care through the end of data collection (48 weeks) or patient death.

The PNC will interact with the patient and caregiver over time and across settings (home, clinic, hospital, and hospice) via telephone using the manualized curriculum: *"Charting Your Course (CYC): An Intervention for Patients with Heart Failure and their Families".* (Table 2 and Appendix B). The CYC materials will be mailed prior to the first session. Participants will be asked to review each chapter prior to the session, but this is not required. Based on our prior work and recent pilot study,[68] sessions last approximately 30-40 minutes.[50] The PNC conducts the scheduled weekly sessions and then continues to follow participants monthly to check on patient/caregiver needs and to reinforce prior content. PCT and PNC involvement is complementary; the in-person PCT consultation provides expert symptom assessment and builds a foundation for future consultation if needed and the PNC sessions provide comprehensive information in an unhurried, convenient home setting. The primary goal of the intervention is to encourage patient empowerment; however occasionally the PNC may provide feedback directly to the HF (or PCT) teams about specific issues (e.g., unrelieved pain) or make referrals to other resources. The HF and PCT teams are responsible for direct medical care. If an intervention patient is hospitalized they will be followed by the attending service and PCT consultation will be offered.

Table 2. Charting Your Course (CYC) Patient (PT) and Caregiver (CG) Chapter/Session Topics by Specific Aim

Specific Aim 1
**In-person Palliative Care Team (PCT) Consultation following National Consensus Guidelines**:<br>➢Understanding of illness, tx plan, prognosis ➢Decision style and preferences ➢Goals of care ➢Physical symptoms ➢Social hx ➢Support system and challenges ➢Psycho-emotional well-being ➢Spirituality ➢Advance care planning ➢Pharmacological assessment and recommendations ➢Referrals ➢Communicate with PCP ➢Follow-up
**PT CYC Chapter/Session 1:** *Problem Solving and the "COPE" Attitude*<br>➢Distress Thermometer ➢What is problem solving coping? ➢COPE: A Positive Problem-Solving Attitude ➢"C"-Creativity ➢"O"-Optimism ➢"P"-Planning ➢"E"-Expert Information ➢The Seven Steps of Problem-Solving ➢Identifying the Problem ➢Establishing a Goal ➢Brainstorming Solutions ➢Pros and Cons ➢Picking and Implementing a Solution ➢Reviewing how Your Plan Worked ➢ACTIVITY-Giving the 7 Steps a Shot
**PT CYC Chapter/Session 2:** *Self-Care*<br>➢Distress Thermometer ➢What is Self-Care? ➢Healthy Eating and Nutrition ➢Exercise ➢Smoking ➢Relaxation Techniques ➢ACTIVITY-Identifying a Support Team ➢ACTIVITY-Building a Support Team ➢What's the Latest?
**PT CYC Chapter/Session 3:** *Symptom Management*

➢Distress Thermometer ➢Prioritizing and Tracking Symptoms ➢Physical Symptoms of HF ➢"Advanced Therapies" in HF ➢Emotional Effects of HF (Depression, Anxiety, Grief & Loss) ➢ACTIVITY-Identifying Strengths and Resources ➢Spirituality

PT CYC Chapter/Session 4: *Core Values, Talking about what Matters Most, & Making Decisions for the Future*
➢Distress Thermometer ➢Core Values and Decision-Making ➢ACTIVITY-Thinking About My Own Core Values ➢How to Talk to Your Family and Health Care Team about What Matters Most to You ➢Decision Aids ➢ACTIVITY-Watching the Looking Ahead DVD ➢ACTIVITY-The Ottawa Personal Decision Guide ➢Advance Directives

PT CYC Chapter/Session 5: *My Life Story*
➢My Life Story and Why Thinking about it is Important (even if I'm not dying) ➢Where I was born and early childhood ➢Influential people ➢Important events ➢Cherished times ➢Important goals ➢Favorite accomplishments/proudest moments ➢Important elements if there was a movie about your life

PT CYC Chapter/Session 6: *Looking at Today, Looking at Tomorrow*
➢Distress Thermometer ➢What is Forgiveness ➢Regrets ➢People they want to forgive ➢Seeking forgiveness from others ➢Things left undone ➢Feeling at peace ➢Family and personal values ➢Hopes and fears ➢Sources of strength ➢Wisdom gained & lessons learned ➢Advice for future generations ➢Future things to accomplish

Monthly Follow-up:
➢ On-going psychosocial support ➢Reinforce/clarify previous material as it impacts real time decision making, symptom management and QOL.

Specific Aim 2

CG CYC Chapter/Session 1: Mirrors PT Session 1 with special attention to CG role

CG CYC Chapter/Session 2: Mirrors PT Session 2

CG CYC Chapter/Session 3: Mirrors PT Session 3 with special attention to surrogate decision-making role

CG CYC Chapter/Session 4: Mirrors PT Session 4

Monthly Follow-up: Mirrors PT.

Bereavement Follow-up: If PT dies during study period, the PNC contacts the CG for bereavement follow-up

Usual Care

UAB: HF patients randomized to the usual care group will receive their outpatient care through the Advanced HF or Cardiology Clinics (both located at the Kirklin Clinic), or Geriatric HF Clinic (located at UAB Highlands). Primary care practices are located in Birmingham and patients' local communities. Typical HF patient medical management is based on national HF guidelines.[38] Though advanced HF patients may also receive chronic inotropes or mechanical circulatory support devices. Hospitalized patients are managed by the admitting service (Cardiology or Hospitalist). PCT consultation is rare, but may be offered at the discretion of the clinician. In FY 2012-13, UAB PCT consults in HF were: 24 outpatient; 298 inpatient, and 264 PCCU admissions. While most patients receiving advanced therapies (ventricular assist devices [VAD] or transplant) receive a PCT consult as part of their evaluation; these candidates are rarely age 65 and older.
BVAMC: HF patients are followed by primary care, general cardiology and geriatrics with care similar to that described above. In FY 2012-13 HF comprised 20% of outpatient and inpatient consults, Safe Harbor Palliative Care Unit admissions, and 39 HF home hospice admissions.

C3.1 General Study Procedures

Following eligibility screening, signed informed consent, baseline data collection, and randomization, the PNC will contact intervention patients and (enrolled caregivers) to schedule the first CYC phone session (described above). Patient and caregiver CYC Session 1 will occur during the week following enrollment. The remaining sessions will be scheduled for subsequent consecutive weeks. After completing the structured sessions, the PNC will continue phone contact at least monthly. If the patient dies during the study a bereavement call will be made to a (study or non-study) caregiver. After completing the baseline questionnaires upon enrollment, intervention and usual care participants will be contacted by research coordinators (who are blinded to study group) for scheduled, phone-based data collection at 8 weeks after enrollment and every 8 weeks thereafter for 48 weeks.

C3.2 Training and Treatment Fidelity Monitoring

We anticipate hiring PNCs with palliative care, cardiac, and/or psychiatric nursing experience and have commitments from three PNCs who have been providing this intervention during our pilot. These PNCs have received (or additional new PNCs will receive) training provided by study staff (Bakitas/Dionne-Odom/Burgio) which includes 28 hours of didactic and interactive role-play guided by an established training manual/protocol including specific intervention skills (e.g. problem solving, decision support, health coaching). Bakitas/Dionne-Odom/Burgio will review digitally-recorded mock training sessions and provide feedback until PNCs are confident in their skills. (See Nurse Coach Training scripts-Appendix C and Fidelity checklists - Appendix D).

Fidelity Monitoring: Treatment Integrity: PCT Consultation: Co-Is Drs. Kvale and Bailey will review PCT notes which are recorded in the electronic medical record using a template comprised of PCT elements based on NCP Guidelines.[36] If elements are consistently missing, the PCT member will receive additional training.

PNC coaching sessions: The PI and Drs. Burgio and Dionne-Odom will review a random sample of all digitally-recorded coaching sessions. The sessions will be scored using previously developed fidelity checklists that have been adapted to this study (see Fidelity Checklists-Appendix D.). The goal of the fidelity monitoring is to insure that the intervention is being administered reliably over time and across PNCs. PNCs who exhibit a pattern of non-adherence on three consecutive ratings will receive additional training and supervision.

Participant receipt / enactment of intervention: Following the collection of outcome measures at week 48, all intervention participants will be interviewed by phone for the primary purpose of determining if the patient received and enacted the components of the intervention as described in Table 2. We will use procedures and interview guides that have been adapted from our previous RCTs.[67] Briefly we will digitally-record, transcribe all interviews verbatim, and code transcripts using ATLAS.ti software to determine the extent of patient receipt and enactment of the skills taught by the nurse coaches. The PI is an experienced qualitative researcher,[170-174] with expertise in the qualitative techniques that are recommended for intervention evaluation.[175,176] Though not the purpose in this proposal, these interviews may also provide rich data for a secondary analysis of the patient and caregiver experience.

C4 INSTRUMENTS (All instruments are contained in Appendix E.)

The instruments were carefully chosen to measure distinct constructs within the theoretical models (Figures 1a and 1b) with little to no overlap. The proposed instrument battery and schedule are similar to what we have used in our prior studies[50,51,177] and in our recent HF pilot study.[68] Per pilot participants' report[68] these presented minimal burden.

Table 3. Outcome measures

	Construct	Instrument	Description	Reliability	Schedule
**Specific Aim 1**					
HF Patients	**QOL**	Kansas City Cardiomyopathy Questionnaire (KCCQ)[178]	5 domains: physical limitations, symptoms, self-efficacy, social interference, and QOL; 23 items	Subscales α=.62 to .90	Baseline, week 8 and every 8 weeks until week 48
HF Patients	**Mood**	Hospital Anxiety and Depression Scale (HADS)[179]	2 domains measuring depression and anxiety; 14 items	Subscales α=.82 to .83	
HF Patients	**Symptom Burden**	Memorial Symptom Assessment Scale-HF (MSAS-HF)[25]	Targets 32 HF-relevant symptoms (e.g. chest pain, difficulty breathing while lying flat, etc.)	Subscales α=.73 to .92	
HF Patients	**Resource use**	Resource Use	Inpatient days, ICU days, ED visits, hospice use, palliative care provider visits, AD completion, DNR orders	NA	
**Specific Aim 2**					
Caregivers*	**QOL**	Quality of Life – Caregiver (QOLC)[180]	3 domains: emotional and spiritual well-being; relationship with patient; & sleep, daily routine, and family life, 35 items	α = 0.91	Baseline, week 8 and every 8 weeks until week 48
Caregivers*	**Mood**	Hospital Anxiety and Depression Scale (HADS)	Same as above	Same as above	
Caregivers*	**Caregiver burden**	Montgomery Borgatta Caregiver Burden Scale (MBCB)[181]	Measure of caregiver burden with 3 domains: objective burden, stress burden, and demand burden; 14 items	Subscales α = .75 to .88	
Caregivers*	**Self-reported health**	PROMIS SF Global Health[10]	2 domains: physical and mental health, 10 items	Subscales α= .81 to .86	
**Exploratory Aims**					
HF Patients	**Patient Activation**	Patient Assessment of Chronic Illness Care (PACIC)[182]	5 dimensions: activation, delivery system/decision support, goal setting, problem solving, and coordination; 20 items	Subscales α=.62 to .90	Baseline, week 8 and every 8 weeks until week 48
HF Patients	**Coping style**	Brief Cope	2 subscales: active and avoidant coping; 28 items[183]	α = .68 to .79	Baseline only
HF Patients	**Social support**	Multidimensional Scale of Perceived Social Support (MSPSS)	Perceived adequacy of support from family and friends, 12 items[184]	α =.81	
All	**Health literacy**	Newest Vital Sign[9]	Measure of health literacy and numeracy based on interpretations of a nutrition label, 6 items	α = .76	
All	**Demo-graphics**	Demographic questionnaire	Age, gender, race, marital status, religion, employment, education, occupation, health insurance, smoking, etc.	NA	

Additional measures and data

If patients die on study we will conduct a standardized interview with a study or non-study (if agreeable) caregiver 2 months after death using the instruments and similar techniques from prior studies[129,185] to evaluate the caregivers' perception of the EOL care provided and to assess their grief experience and mood. The questionnaires are: 1) proxy-reported Quality of Death and Dying (QODD) (31-items) which rates the quality of the descendant's last 7 days; it has acceptable internal consistency (Cronbach alphas 0.89) and construct validity; [186,187] 2) Inventory of Complicated Grief-Short form (ICG-SF) a reliable, validated 13-item survey of complicated grief[188] and the HADs (described above).

C4.1 Data Collection, Management, and Quality Control.

Participant recruitment/ tracking: A Microsoft Access database will be used to track the participant progress through recruitment, enrollment, and all study procedures. The database will consist of 3 sections: a form with contact information about interested participants (e.g., name, current address, cell and telephone numbers, and the best time to call); a section allowing for tracking contact attempts for potential participants; a dates form to document the number and outcomes of participant contacts (i.e., scheduled or enrolled), and all

subsequent contact times and types, specific mailed contacts, dates and times of pertinent communication, and information about the length of time between scheduled visits and dates of missed appointments.

Data collection of outcome measures: Several instruments are available for completion within the electronic health record. We are exploring the ability to collect most of the study data via this mechanism. In the event this is not feasible we will use our trained research coordinators to collect patient- and caregiver-reported data by phone and it will be directly-entered into our existing REDCap (Research Electronic Data Capture) database. REDCap is software for building and managing questionnaires and facilitating electronic data collection and storage. It supports a HIPAA best practice, secure web-based application enabling multiple sites to seamlessly access, analyze and share data while maintaining the security and integrity of the database and provides automated export procedures for seamless data downloads to common statistical packages (SPSS, SAS), built-in project calendars, scheduling and randomization modules. Hard stops prevent missing data due to inadvertent skipping of items.

eHR and administrative data collection: Resource use, advance directives, use of palliative, hospice, and home care services, disease stage, and other clinical data will be collected using established data collection forms and chart abstraction techniques/protocols that we have used previously.[63,68,158] (See Appendix E for forms).

Data quality control (interviewing and chart abstraction): We will hire experienced research coordinators who have received intensive training (typically 10 hours including role play and inter-rater reliability checks) in order to assure high quality data collection from participants and electronic medical records.

Data Security**:** All databases are secure, HIPPA-compliant and password protected in both the front end and back end. Back end data will be housed on a secure drive with access limited to only authorized research personnel.

C5 STATISTICAL CONSIDERATIONS

C5.1 Randomization. After consent and baseline assessment, patients will be randomized using computer-generated treatment assignments, stratified by center (UAB or BVAMC), referring service (HF clinic, cardiology, primary care), and race (Caucasian/non-Caucasian).

C5.2 Statistical Analyses

Specific Aim 1 Patient-reported QOL, mood, symptom burden, and resource use

The intention to treat (ITT) analysis will be used for all treatment comparisons. That is, all randomized patients will be included in primary comparisons, regardless of whether the patient actually received the randomly-assigned intervention. Primary data analysis will begin with descriptive statistics for baseline patient characteristics and outcomes by treatment group. Distributional assumptions will be examined; when appropriate we will employ testing and modeling procedures that do not assume normality. We will compare groups with respect to baseline covariates. We will analyze and compare missing data and compliance patterns according to baseline covariates and we will adjust analyses for baseline factors showing either imbalances or predictive of missing data (not due to patient death) or compliance. Mixed modeling techniques and covariate adjustment will reduce the impact of missing data, provided that the data are missing at random. If necessary, multiple imputation techniques and sensitivity analyses will be used to deal with data not missing at random and not caused by patient death.

We will conduct 2 sets of longitudinal ITT analyses of the primary study endpoints for participants with baseline and one or more follow up assessments using mixed effects modeling for repeated measures to examine the relative impact of ENABLE: CHF-PC in the 48 weeks after enrollment (forward in time from baseline), and its effects proximal to the time to death (retrospective starting from death). In the first set of analyses, we will examine differences between all patients in each group surviving until the scheduled 48 week follow-up surveys. Kurland et al.[147] have described this approach as a partially conditional analysis for joint longitudinal and survival data, because it assumes that patients are alive at the comparison times.[147] Initial ITT analyses will be conducted on patient-reported outcomes using mixed effects models to test the effect of the intervention, first by comparing all group differences simultaneously for both time periods in multiple degree of freedom global tests, and then individually if overall differences are significant.

The second analysis, retrospective from death, also conducted with mixed effects models, has been described by Kurland et al.[147] as fully conditional; because it examines patient outcomes conditional on the patients having died (the participants who are alive by the end of follow-up are excluded because their outcome measures proximal to death will be unknown). Alternative more complex analyses involving “terminal decline” models that jointly model a longitudinal outcome and survival will be considered as described under Exploratory Aim 2 below. All calculations will be performed using the latest versions of SAS and R. Standardized intervention effect sizes will be estimated for each outcome, and the False Discovery Rate criterion will be used to correct for multiple significance testing.

We will conduct a decedent-only ITT analysis for the proxy-report QODD. Since only one QODD observation will be available for each decedent, no longitudinal modeling is required. Secondary analyses will consider any deviations from the intended intervention, and intervention dose effects. Subgroup analyses will be conducted with interest on

determining the profile characteristics (e.g. race, SES, gender, military vs. civilian background, absence of caregiver, etc.) of the participants who benefited the most and the least from the intervention using flexible modeling techniques, such as latent class analysis and recursive partitioning.

Specific Aim 2 Caregiver QOL, mood, self-reported health, burden

Caregiver outcomes will be analyzed using the same longitudinal methods as outlined for Specific Aim 1. For the analyses going forward in time from baseline, the effects of patient death on caregiver outcomes will be examined using models with indicators for patient death at its respective time-point. Only decedents' caregivers will have grief outcomes analyzed which will not involve longitudinal modeling. Caregivers of patients who die will contribute the additional outcome of a rating of quality of care at the end of life (QODD). Since only one QODD observation will be available for each decedent, no longitudinal modeling is required. However, analysis of these measures will be conducted with mixed effect methods to account for nesting, with the parameter of interest being the association between group assignment and the caregiver-rated outcome

Exploratory Aim 1. Explore mediators/moderators of patient/caregiver outcomes and reciprocal relationships.

As depicted earlier in Figure 1a, intervention effect on patient QOL, mood, and symptom burden is proposed to be mediated by the quality of chronic illness care (PACIC), active and avoidant coping styles (COPE), social support (MSPSS) and health literacy (NVS). This conceptual logic will be tested using structural equation modeling (SEM) applied to matrices residualized for demographic and disease factors.[189] Each patient model will involve 13 observed variables: the intervention as a dichotomous variable, five mediational variables (8 week: PACIC, active COPE, avoidant COPE, MSPSS, NVS) lagged against their five baseline values, and the 8 week outcome of interest lagged against its baseline. With 22 degrees of freedom, a sample size of 359 (expected to be alive/available at week 8), and a significance level of p= .05, power is .86 to distinguish a model with close fit (root mean square error of approximation [RMSEA] = .05) from a model with mediocre to poor fit (RMSEA = .09).[190] Jointly significant effects involving the theorized mediators (intervention → mediator, mediator → outcome as depicted in Figure 1a) will constitute evidence of mediation. Assuming a medium effect size (d=0.5) on mediator and outcome, a sample size of 359 provides.80 power to detect a small indirect effect of at least 34% of the total intervention effect size (d_M=0.17) based on computer simulations of joint tests with a significance level of 0.05.[191]

As depicted earlier in Figure 1b, the effect of the intervention on caregiver QOL, mood, and burden is proposed to be mediated by patient well-being. Tests of mediation of caregiver outcomes will involve jointly significant (p = .05) effects (intervention → 8 week patient outcome → 24 week caregiver outcome) for caregiver outcomes mediated by patient outcome. Assuming a medium effect size (d=0.5) on both mediator and outcome, a sample size of 188 (expected CGs with 24 week outcomes) provides .80 power to detect an indirect effect size of at least 48% of the total intervention effect size (d_M=0.24) based on computer simulations of joint tests with a significance level of 0.05.[191] As depicted earlier in Figure 1b, the effect of the intervention on caregiver QOL, mood, and burden is proposed to be mediated by patient well-being. Tests of mediation of caregiver outcomes will involve jointly significant (p = .05) effects (intervention → 8 week patient outcome → 24 week caregiver outcome) for caregiver outcomes mediated by patient outcome. Assuming a medium effect size (d=0.5) on both mediator and outcome, a sample size of 188 (expected CGs with 24 week outcomes) provides .80 power to detect an indirect effect size of at least 48% of the total intervention effect size (d_M=0.24) based on computer simulations of joint tests with a significance level of 0.05.[191]

Exploratory Aim 2. Examine intervention effects using joint modeling approaches.

Previously cited palliative care studies have shown alterations in both QOL and survival.[63,153] Although the interventions are not directly intended to impact survival, evaluation of this effect is important. We will compare survival between groups using Kaplan-Meier plots and log-rank tests. Relative risks and differences in median survival times will be estimated and confidence intervals will be constructed. For a second set (assessment prior to death) of analyses, we will implement a joint analysis of the survival time and the longitudinal data. The likelihood for this model is constructed by specifying the survival distribution as a piecewise exponential distribution depending on the group assignment, and the outcomes before death as longitudinal outcomes from mixed models depending on time measuring backwards from death. Kurland et al.[147] describe this as the fully conditional terminal decline approach to joint modeling. [63,153]. To accommodate the intermittent decline, flexible spline-based time trends will be introduced in the terminal decline model.[146] With this model, we will be able to construct estimates of mean survival time in each intervention group weighted by longitudinal QOL measures. These estimates will allow comparison between the groups in terms of cumulative QOL factoring in any differences in survival time.

C5.3 Sample Size Considerations Assuming a projected uniform patient accrual rate over 42 months we anticipate an initial enrollment of 380 patients and

Table 4. MDD for patient outcomes between arms based on longitudinal analysis of patients alive at follow up to 48 weeks.

F/U (wks)	No. Alive/ Available	KCCQ (sd=21)	MSAS-HF (sd=2.01)	HADS-A (sd=4.0)	HADS-D (sd=3.9)
8	356	6.6	0.64	1.26	1.23
16	334	6.41	0.62	1.22	1.19
24	313	6.41	0.62	1.22	1.19
32	294	6.49	0.63	1.24	1.21
40	276	6.6	0.64	1.26	1.23
48	259	6.72	0.65	1.28	[illegible]

Table 5. MDD between arms based on longitudinal analysis of CG available at follow-up times up to 48 weeks.

F/U (wks)	Number Available	C-QOL (sd=18.6)	MBCB-OB (sd=3.66)
8	214	107	0.405
16	201	101	0.393
24	188	94	0.394
32	176	88	0.399
40	165	83	0.405
48	155	78	0.414

228 caregivers. We assume an exponential survival distribution with a median of 2 years and independent 2-year 10% attrition. Under these assumptions, we estimate the minimum detectable difference (MDD) between the intervention and control patient means at every 8 weeks post randomization for 48 weeks as described in Table 4. We apply this same method to caregivers (Table 5) who will have their follow-ups truncated at patient death. We use ENABLE II data to provide estimates for the caregiver MBCB-OB standard deviation (sd).[129] Based on the meta-analysis by Garin et al.[192] Literature-based estimates are used for the KCCQ,[193] HADS,[179,194] MSAS-HF,[25] and QOL-C.[104] Patient and caregiver MDDs shown in Table 4 and 5 are based on partially-conditional (on being alive) time-averaged tests of mean differences comparing the treatment groups at the specified follow-up times, intra-subject correlation of 0.5, with a corrected two-sided .01 significance level, and a power of 0.80. Under these conservative assumptions we have sufficient sample size for the primary aims of this study. For subgroup analyses (e.g. Caucasian race vs. other) the detectable time-averaged standardized difference in intervention effect by 48 weeks post randomization is 0.53, assuming equally sized subgroups, and prior to other covariate adjustment.

It is estimated that 120 patients (60 per group) will die by 48 weeks post-randomization and will be available for the analysis of patient outcomes nearest death. Based on the sds in Table 4, we find MDDs of 10.8 for KCCQ, 1.04 for MSAS-HF, 2.06 for HADS-A, and 2.01 for HADS-D. We project that the grief index (ICG) will be available for 37 caregivers in each group. With a sd of 11.7,[63] a 5% two-sided significance level and 80% power, the MDD of the ICG between two groups is 7.7 using a two-sample t-test.

For secondary comparison of survival outcomes, we make the same assumptions about median survival, attrition, accrual, and observation time as for the longitudinal outcomes. Based on a log rank test, .05 significance level and a power of .80, a 1-yr reference survival fraction of 0.68, the detectable HR is 1.6.

C6 Potential problems, alternative strategies, and benchmarks for success and future directions.

We have anticipated potential problems and solutions related to sample recruitment and retention, usual care group contamination, feasibility, non-participation and respondent bias. Recruitment and retention is expected to be robust based on the strong collaborative relationships between the HF and palliative care clinical and research teams and the availability of a large existing HF cohort. Research coordinators will be present in all HF clinical areas. The PI and co-PIs will meet regularly to track recruitment and review issues and jointly problem solve. While usual care group contamination is possible because HF clinicians are seeing intervention and usual care participants; early palliative care referral of HF patients at the UAB/BVAMC a very new concept[22,39] as evidenced by the current negligible HF patients being seen in consultation. Our intervention is not specifically directed at changing physician behavior. Prior studies have shown that simply exposing physicians to new practices and providing education are not sufficient to change physician behavior[69] so while control group contamination due to physician behavior change is possible, it is extremely unlikely.[23] Feasibility is most directly addressed based on our prior successful completion of similar studies. Our team collectively has conducted numerous large, complex, multi-site RCTs in a rigorous fashion over the past decade. UAB/BVAMC is a research intensive environment (See Facilities/Resources) providing an exceptional infrastructure within which to conduct this study successfully. Non-participation bias will be monitored by collecting demographic data on person's who decline and comparing that to patients who participate. We have established tracking and data collection mechanisms to capture this data carefully. Respondent bias in a non-blinded study is an important concern that we will address by providing similar information about the study purpose generally to both arms but not the specific outcomes we wish to influence and also by blinding data collectors to study group.

C7 Study Timeline and Milestones

As a result of our 10 years of experience conducting similar palliative care clinical trials our established investigators recruitment, retention, and data collection methods, we anticipate prompt regulatory approval (within 3 months of funding) and study start-up. This will ensure a 42-month recruitment period and an additional 6 months of follow up on patient and caregiver (bereavement) outcomes. We will present yearly progress reports to the Data Safety Monitoring Committee for feedback on study progress and to identify solutions to any unforeseen challenges or adverse events.

	Year 1				Year 2				Year 3				Year 4				Year 5			
**Activity**	Q 1	Q2	Q3	Q4	Q 1	Q2	Q3	Q4	Q 1	Q2	Q3	Q4	Q 1	Q2	Q3	Q4	Q 1	Q2	Q3	Q4
**Aim 1 & 2**																				
Hire, train staff, IRB approval																				
Enrollment																				
Collect Data																				
**Data Analysis (exploratory aims (1&2)**																				
Progress/Final Reports Grant submission																				

REFERENCES

1. Go AS, Mozaffarian D, Roger VL, et al. Heart disease and stroke statistics--2014 update: a report from the american heart association. *Circulation.* Jan 2014;129(3):e28-e292.
2. Liu L, Eisen HJ. Epidemiology of Heart Failure and Scope of the Problem. *Cardiology Clinics.* 2// 2014;32(1):1-8.
3. Stein GY, Kremer A, Shochat T, et al. The diversity of heart failure in a hospitalized population: the role of age. *J Card Fail.* Aug 2012;18(8):645-653.
4. Will JC, Valderrama AL, Yoon PW. Preventable hospitalizations for congestive heart failure: establishing a baseline to monitor trends and disparities. *Preventing chronic disease.* 2012;9:E85.
5. Robinson CA, Pesut B, Bottorff JL, Mowry A, Broughton S, Fyles G. Rural Palliative Care: A Comprehensive Review. *J Palliat Med.* Feb 13 2009.
6. Bekelman DB, Hutt E, Masoudi FA, Kutner JS, Rumsfeld JS. Defining the role of palliative care in older adults with heart failure. *International journal of cardiology.* Apr 10 2008;125(2):183-190.
7. Buck HG, Riegel B. The impact of frailty on health related quality of life in heart failure. *European journal of cardiovascular nursing : journal of the Working Group on Cardiovascular Nursing of the European Society of Cardiology.* Jun 28 2010.
8. Waterworth S, Jorgensen D. It's not just about heart failure--voices of older people in transition to dependence and death. *Health Soc Care Community.* Mar 2010;18(2):199-207.
9. Shah KB, Rahim S, Boxer RS. Heart Failure Readmissions. *Current treatment options in cardiovascular medicine.* Jul 2 2013.
10. Unroe KT, Greiner MA, Hernandez AF, et al. Resource use in the last 6 months of life among medicare beneficiaries with heart failure, 2000-2007. *Arch Intern Med.* Feb 14 2011;171(3):196-203.
11. Bekelman DB, Rumsfeld JS, Havranek EP, et al. Symptom burden, depression, and spiritual well-being: a comparison of heart failure and advanced cancer patients. *Journal of general internal medicine.* May 2009;24(5):592-598.
12. Currow DC, Smith J, Davidson PM, Newton PJ, Agar MR, Abernethy AP. Do the trajectories of dyspnea differ in prevalence and intensity by diagnosis at the end of life? A consecutive cohort study. *J Pain Symptom Manage.* Apr 2010;39(4):680-690.
13. Goebel JR, Doering LV, Lorenz KA, Maliski SL, Nyamathi AM, Evangelista LS. Caring for special populations: total pain theory in advanced heart failure: applications to research and practice. *Nursing forum.* Jul-Sep 2009;44(3):175-185.
14. Goebel JR, Doering LV, Shugarman LR, et al. Heart failure: the hidden problem of pain. *J Pain Symptom Manage.* Nov 2009;38(5):698-707.
15. Goodlin SJ. Palliative care in congestive heart failure. *Journal of the American College of Cardiology.* Jul 28 2009;54(5):386-396.
16. O'Leary N, Murphy NF, O'Loughlin C, Tiernan E, McDonald K. A comparative study of the palliative care needs of heart failure and cancer patients. *Eur J Heart Fail.* Apr 2009;11(4):406-412.
17. Fang J, Mensah GA, Croft JB, Keenan NL. Heart failure-related hospitalization in the U.S., 1979 to 2004. *Journal of the American College of Cardiology.* Aug 5 2008;52(6):428-434.
18. Evangelista LS, Liao S, Motie M, De Michelis N, Lombardo D. On-going palliative care enhances perceived control and patient activation and reduces symptom distress in patients with symptomatic heart failure: A pilot study. *European journal of cardiovascular nursing : journal of the Working Group on Cardiovascular Nursing of the European Society of Cardiology.* Jan 17 2014.
19. Evangelista LS, Liao S, Motie M, De Michelis N, Ballard-Hernandez J, Lombardo D. Does the type and frequency of palliative care services received by patients with advanced heart failure impact symptom burden? *J Palliat Med.* Jan 2014;17(1):75-79.
20. Evangelista LS, Lombardo D, Malik S, Ballard-Hernandez J, Motie M, Liao S. Examining the effects of an outpatient palliative care consultation on symptom burden, depression, and quality of life in patients with symptomatic heart failure. *J Card Fail.* Dec 2012;18(12):894-899.
21. Bekelman DB, Nowels CT, Allen LA, Shakar S, Kutner JS, Matlock DD. Outpatient palliative care for chronic heart failure: a case series. *J Palliat Med.* Jul 2011;14(7):815-821.
22. Yancy CW, Jessup M, Bozkurt B, et al. 2013 ACCF/AHA Guideline for the Management of Heart Failure: A Report of the American College of Cardiology Foundation/American Heart Association Task Force on Practice Guidelines. *Journal of the American College of Cardiology.* 10/15/ 2013;62(16):e147-e239.

23. Evangelista LS, Motie M, Lombardo D, Ballard-Hernandez J, Malik S, Liao S. Does preparedness planning improve attitudes and completion of advance directives in patients with symptomatic heart failure? *J Palliat Med.* Dec 2012;15(12):1316-1320.
24. Fitzsimons D, Strachan PH. Overcoming the challenges of conducting research with people who have advanced heart failure and palliative care needs. *European journal of cardiovascular nursing : journal of the Working Group on Cardiovascular Nursing of the European Society of Cardiology.* Feb 15 2011.
25. Bekelman DB, Nowels CT, Retrum JH, et al. Giving voice to patients' and family caregivers' needs in chronic heart failure: implications for palliative care programs. *J Palliat Med.* Dec 2011;14(12):1317-1324.
26. Thai V, Cujec B. Transitioning to end-of-life care for patients with advanced heart failure. *J Palliat Med.* Jul 2010;13(7):796.
27. Janssen DJ, Spruit MA, Schols JM, Wouters EF. A call for high-quality advance care planning in outpatients with severe COPD or chronic heart failure. *Chest.* Sep 9 2010.
28. Davidson PM, Macdonald PS, Newton PJ, Currow DC. End stage heart failure patients - palliative care in general practice. *Aust Fam Physician.* Dec 2010;39(12):916-920.
29. Berry JI. Hospice and heart disease: missed opportunities. *J Pain Palliat Care Pharmacother.* Mar 2010;24(1):23-26.
30. Widera E, Pantilat SZ. Hospitalization as an opportunity to integrate palliative care in heart failure management. *Curr Opin Support Palliat Care.* Dec 2009;3(4):247-251.
31. Chattoo S, Atkin KM. Extending specialist palliative care to people with heart failure: semantic, historical and practical limitations to policy guidelines. *Soc Sci Med.* Jul 2009;69(2):147-153.
32. Howlett JG. Palliative care in heart failure: addressing the largest care gap. *Curr Opin Cardiol.* Mar 2011;26(2):144-148.
33. Hupcey JE, Penrod J, Fogg J. Heart failure and palliative care: implications in practice. *J Palliat Med.* Jun 2009;12(6):531-536.
34. Bakitas M, Bishop MF, Caron P, Stephens L. Developing successful models of cancer palliative care services. *Semin Oncol Nurs.* Nov 2010;26(4):266-284. PMID: 20971407: PMCID PMC2976669
35. Bakitas M, Lyons K, Hegel M, et al. Effects of a palliative care intervention on clinical outcomes in patients with advanced cancer: The Project ENABLE II Randomized Controlled Trial. *JAMA.* 2009;302(7):741-749. PMID: 19690306; PMCID: PMC3657724
36. El-jawahri A, Greer JA, Temel JS. Does Palliative Care Improve Outcomes for Patients with Incurable Illness? A Review of the Evidence. *The Journal of Supportive Oncology.* 2011;9(3):87-94.
37. Temel JS, Greer JA, Muzikansky A, et al. Early palliative care for patients with metastatic non-small-cell lung cancer. *The New England journal of medicine.* Aug 19 2010;363(8):733-742.
38. Bakitas M, Ahles TA, Skalla K, et al. Proxy perspectives regarding end-of-life care for persons with cancer. *Cancer.* Apr 15 2008;112(8):1854-1861. PMID: 18306393; PMCID: 3638939
39. Bakitas M, Lyons K, Hegel M, et al. Project ENABLE II randomized controlled trial to improve palliative care for rural patients with advanced cancer: Baseline findings, methodological challenges, and solutions. *Palliat Support Care.* 2009;7:75-86. PMID: 19619377; PMCID: PMC3685415
40. Bakitas M, Stevens M, Ahles T, et al. Project ENABLE: A palliative care demonstration project for advanced cancer patients in three settings. *J Palliat Med.* 2004;7(2):363-372.
41. Smith TJ, Temin S, Alesi ER, et al. American Society of Clinical Oncology Provisional Clinical Opinion: The Integration of Palliative Care into Standard Oncology Care. *J Clin Oncol.* Feb 6 2012.
42. Gomes B, Calanzani N, Curiale V, McCrone P, Higginson IJ. *Effectiveness and cost-effectiveness of home palliative care services for adults with advanced illness and their caregivers.* London2013. Issue 6. Art.No.:CD007760.
43. World Health Organization. WHO Definition of Palliative Care 2010; http://www.who.int/cancer/palliative/definition/en/. Accessed November, 2010.
44. Dionne-Odom JN, Kono; A., Ahmed A, et al. Translating and Testing a Concurrent Palliative Care Model for Rural-Dwelling Adults with Heart Failure and their Family Caregivers: ENABLE: CHF-PC. *J Palliat Med.* 2014, in press.
45. Weiss BD, Mays MZ, Martz W, et al. Quick assessment of literacy in primary care: the newest vital sign. *Ann Fam Med.* 2005 Nov-Dec 2005;3(6):514-522.
46. Durant RW, Brown QL, Cherrington AL, Andreae LJ, Hardy CM, Scarinci IC. Social support among African Americans with heart failure: is there a role for community health advisors? *Heart Lung.* Jan-Feb 2013;42(1):19-25.

47. Li Z, Tosteson TD, Bakitas MA. Joint modeling quality of life and survival using a terminal decline model in palliative care studies. *Statistics in medicine.* Apr 15 2013;32(8):1394-1406 PMID: 23001893; PMCID: PMC3623280
48. Williams BR, Woodby LL, Bailey FA, Burgio KL. Formative Evaluation of a Multi-Component, Education-Based Intervention to Improve Processes of End-of-Life Care. *Gerontology & geriatrics education.* 2014;35(1):4-22.
49. Bailey FA, Williams BR, Woodby LL, et al. Intervention to Improve Care at Life's End in Inpatient Settings: The BEACON Trial. *Journal of general internal medicine.* Jan 22 2014.
50. Leigh AE, Burgio KL, Williams BR, Kvale E, Bailey FA. Hospice emergency kit for veterans: a pilot study. *J Palliat Med.* Apr 2013;16(4):356-361.
51. Casarett D, Pickard A, Bailey FA, et al. Do palliative consultations improve patient outcomes? *J Am Geriatr Soc.* Apr 2008;56(4):593-599.
52. Bailey FA, Ferguson L, Williams BR, et al. Palliative care intervention for choice and use of opioids in the last hours of life. *J Gerontol A Biol Sci Med Sci.* Sep 2008;63(9):974-978.
53. Bailey FA, Burgio KL, Woodby LL, et al. Improving processes of hospital care during the last hours of life. *Arch Intern Med.* Aug 8-22 2005;165(15):1722-1727.
54. Cheng JW, Nayar M. A review of heart failure management in the elderly population. *Am J Geriatr Pharmacother.* Oct 2009;7(5):233-249.
55. Havranek EP, Masoudi FA, Westfall KA, Wolfe P, Ordin DL, Krumholz HM. Spectrum of heart failure in older patients: results from the National Heart Failure project. *American heart journal.* Mar 2002;143(3):412-417.
56. Martinez-Selles M, Vidan MT, Lopez-Palop R, et al. End-stage heart disease in the elderly. *Rev Esp Cardiol.* Apr 2009;62(4):409-421.
57. Lorenz KA, Lynn J, Dy SM, et al. Evidence for improving palliative care at the end of life: a systematic review. *Ann Intern Med.* Jan 15 2008;148(2):147-159.
58. Zambroski C. Self-care at the end of life in patients with heart failure. *Journal of Cardiovascular Nursing.* May-Jun 2008;23(3):266-276.
59. Zambroski CH, Moser DK, Bhat G, Ziegler C. Impact of symptom prevalence and symptom burden on quality of life in patients with heart failure. *European journal of cardiovascular nursing : journal of the Working Group on Cardiovascular Nursing of the European Society of Cardiology.* Sep 2005;4(3):198-206.
60. Zambroski CH, Moser DK, Roser LP, Heo S, Chung ML. Patients with heart failure who die in hospice. *American heart journal.* Mar 2005;149(3):558-564.
61. Hunt SA, Abraham WT, Chin MH, et al. 2009 focused update incorporated into the ACC/AHA 2005 Guidelines for the Diagnosis and Management of Heart Failure in Adults: a report of the American College of Cardiology Foundation/American Heart Association Task Force on Practice Guidelines: developed in collaboration with the International Society for Heart and Lung Transplantation. *Circulation.* Apr 14 2009;119(14):e391-479.
62. Johnson MJ, Booth S. Palliative and end-of-life care for patients with chronic heart failure and chronic lung disease. *Clin Med.* Jun 2010;10(3):286-289.
63. Steinhauser KE, Arnold RM, Olsen MK, et al. Comparing Three Life-Limiting Diseases: Does Diagnosis Matter or is Sick, Sick? *J Pain Symptom Manage.* Jan 27 2011.
64. Maessen M, Veldink JH, van den Berg LH, Schouten HJ, van der Wal G, Onwuteaka-Philipsen BD. Requests for euthanasia: origin of suffering in ALS, heart failure, and cancer patients. *J Neurol.* Jul 2010;257(7):1192-1198.
65. Goebel JR, Doering LV, Evangelista LS, et al. A comparative study of pain in heart failure and non-heart failure veterans. *J Card Fail.* Feb 2009;15(1):24-30.
66. De Jong MJ, Hall LA. Measurement of anxiety for patients with cardiac disease - A critical review and analysis. *Journal of Cardiovascular Nursing.* Sep-Oct 2006;21(5):412-419.
67. De Jong MJ, Moser DK, Chung ML. Anxiety, depression, and functional status are the best predictors of health status for patients with heart failure. *Journal of Cardiac Failure.* Aug 2004;10(4):S21-S21.
68. De Jong MJ, Chung ML, Wu J-R, Riegel B, Rayens MK, Moser DK. Linkages between anxiety and outcomes in heart failure. *Heart & Lung: The Journal of Acute and Critical Care.* 2011;40(5):393-404.
69. Faller H, Stork S, Schuler M, et al. Depression and disease severity as predictors of health-related quality of life in patients with chronic heart failure--a structural equation modeling approach. *J Card Fail.* May 2009;15(4):286-292 e282.

70. National Consensus Project. *Clinical Practice Guidelines for Quality Palliative Care; Second Edition.* Brooklyn, NY: National Consensus Project for Quality Palliative Care;2009.
71. Lewis EF. End of life care in advanced heart failure. *Current treatment options in cardiovascular medicine.* Feb 2011;13(1):79-89.
72. James T, Offer M, Wilson M, Delyea A, Johnson D. Increasing Palliative Consults for Heart Failure Inpatients Using the Seattle Heart Failure Model. *Journal of Hospice & Palliative Nursing.* Sep-Oct 2010;12(5):273-281.
73. Jaarsma T, Beattie JM, Ryder M, et al. Palliative care in heart failure: a position statement from the palliative care workshop of the Heart Failure Association of the European Society of Cardiology. *Eur J Heart Fail.* May 2009;11(5):433-443.
74. Garin O, Ferrer M, Pont A, et al. Disease-specific health-related quality of life questionnaires for heart failure: a systematic review with meta-analyses. *Qual Life Res.* Feb 2009;18(1):71-85.
75. Kheirbek RE, Alemi F, Citron BA, Afaq MA, Wu H, Fletcher RD. Trajectory of illness for patients with congestive heart failure. *J Palliat Med.* May 2013;16(5):478-484.
76. Hupcey JE, Penrod J, Fenstermacher K. Review article: a model of palliative care for heart failure. *Am J Hosp Palliat Care.* Oct-Nov 2009;26(5):399-404.
77. Konstam MA, Greenberg B. Transforming health care through the medical home: the example of heart failure. *J Card Fail.* Nov 2009;15(9):736-738.
78. Waterworth S, Gott M. Decision making among older people with advanced heart failure as they transition to dependency and death. *Curr Opin Support Palliat Care.* Dec 2010;4(4):238-242.
79. Asch SM, Baker DW, Keesey JW, et al. Does the collaborative model improve care for chronic heart failure? *Med Care.* Jul 2005;43(7):667-675.
80. Rich MW, Beckham V, Wittenberg C, Leven CL, Freedland KE, Carney RM. A multidisciplinary intervention to prevent the readmission of elderly patients with congestive heart failure. *The New England journal of medicine.* Nov 2 1995;333(18):1190-1195.
81. Blue L, Lang E, McMurray JJV, et al. Randomised controlled trial of specialist nurse intervention in heart failure. *BMJ: British Medical Journal.* 2001;323(7315):715.
82. Gonseth J, Guallar-Castillón P, Banegas JR, Rodríguez-Artalejo F. The effectiveness of disease management programmes in reducing hospital re-admission in older patients with heart failure: a systematic review and meta-analysis of published reports. *European Heart Journal.* September 1, 2004 2004;25(18):1570-1595.
83. McAlister FA, Stewart S, Ferrua S, McMurray JJJV. Multidisciplinary strategies for the management of heart failure patients at high risk for admission: A systematic review of randomized trials. *Journal of the American College of Cardiology.* 2004;44(4):810-819.
84. Stewart S, Horowitz JD. Home-Based Intervention in Congestive Heart Failure: Long-Term Implications on Readmission and Survival. *Circulation.* June 18, 2002 2002;105(24):2861-2866.
85. Strömberg A, Mårtensson J, Fridlund B, Levin LÅ, Karlsson J-E, Dahlström U. Nurse-led heart failure clinics improve survival and self-care behaviour in patients with heart failure. *European Heart Journal.* 2003;24(11 %U http://eurheartj.oxfordjournals.org/content/24/11/1014.abstract):1014-1023.
86. Krumholz HM, Amatruda J, Smith GL, et al. Randomized trial of an education and support intervention to prevent readmission of patients with heart failure. *Journal of the American College of Cardiology.* Jan 2 2002;39(1):83-89.
87. Bakitas M, Lyons K, Hegel M, et al. The Project ENABLE II Randomized Controlled Trial to Improve Palliative Care for Patients with Advanced Cancer. Paper presented at: 10th National Conference on Cancer Nursing Research2009; Orlando,FL.
88. Bakitas M, Lyons KD, Hegel MT, et al. The project ENABLE II randomized controlled trial to improve palliative care for rural patients with advanced cancer: Baseline findings, methodological challenges, and solutions. *Palliative and Supportive Care.* 2009;7(01):75-86.
89. McCorkle R, Ercolano E, Lazenby M, et al. Self-Management: Enabling and Empowering Patients Living With Cancer as a Chronic Illness. *Ca-a Cancer Journal for Clinicians.* Jan-Feb 2011;61(1):50-62.
90. Jacobsen J, Jackson V, Dahlin C, et al. Components of Early Outpatient Palliative Care Consultation in Patients with Metastatic Nonsmall Cell Lung Cancer. *J Palliat Med.* Apr 2011;14(4):459-464.
91. Bakitas M, Lyons KD, Hegel MT, Ahles T. Oncologists' perspectives on concurrent palliative care in a National Cancer Institute-designated comprehensive cancer center. *Palliative & supportive care.* Oct 8 2012:1-9.

92. Maloney C, Lyons KD, Li Z, Hegel M, Ahles TA, Bakitas M. Patient perspectives on participation in the ENABLE II randomized controlled trial of a concurrent oncology palliative care intervention: benefits and burdens. *Palliative medicine.* Apr 2013;27(4):375-383 PMID: 22573470; PMCID: PMC3657725.
93. Goldstein N, Bradley E, Zeidman J, Mehta D, Morrison RS. Barriers to conversations about deactivation of implantable defibrillators in seriously ill patients: results of a nationwide survey comparing cardiology specialists to primary care physicians. *Journal of the American College of Cardiology.* Jul 21 2009;54(4):371-373.
94. Goldstein N, Carlson M, Livote E, Kutner JS. Brief communication: Management of implantable cardioverter-defibrillators in hospice: A nationwide survey. *Ann Intern Med.* Mar 2 2010;152(5):296-299.
95. Goldstein NE, Lynn J. Trajectory of end-stage heart failure: the influence of technology and implications for policy change. *Perspect Biol Med.* Winter 2006;49(1):10-18.
96. Morrison R. S. MDE. *America's Care of Serious Illness: A state-by-state report card on access to palliative care in our nation's hospitals.* New York: Center to Advance Palliative Care;2011.
97. Bakitas M, Clifford K, Kvale E. Chapter 43: Rural Palliative Care. In: Ferrell BR, Coyle N, eds. *Oxford Textbook of Palliative Nursing, 4rd Edition*. Oxford: Oxford University Press.; 2014 (in press).
98. Zuniga M, Anderson D, Alexander K. Heart Disease and Stroke in Rural America. In: Gamm L, Hutchison L, Dabney B, Dorsey A, eds. *Rural Healthy People 2010: A Companion Document to Healthy People 2010.* Vol 2. College Station, Texas2010:73-84.
99. United States Department of Agriculture-Economic Research Service. The Economics of Food, Farming, Natural Resources, and Rural America: Measuring Rurality: What is Rural? 2007; http://www.ers.usda.gov/Briefing/Rurality/WhatisRural/. Accessed March 26, 2009.
100. Institute of Medicine. *Crossing the Quality Chasm: A new health system for the 21st century.* Washington, DC: National Academy Press;2001.
101. Center to Advance Palliative Care, National Palliative Care Research Center. *America's Care of Serious Illness: A State-by-State Report Card on Access to Palliative Care in Our Nation's Hospitals.* New York: Center to Advance Palliative Care;2008.
102. McCarthy EP, Burns RB, Davis RB, Phillips RS. Barriers to hospice care among older patients dying with lung and colorectal cancer. *Journal of Clinical Oncology.* 2003;21:728-735.
103. Haller IV, Gessert CE. Utilization of medical services at the end of life in older adults with cognitive impairment: focus on outliers. *Journal of palliative medicine.* Apr 2007;10(2):400-407.
104. Heart Failure Society of America. Health Literacy and Care of the Patient with Heart Failure: A Fact Sheet. St. Paul, MN: Heart Failure Society of America; 2009.
105. Wu JR, Holmes GM, DeWalt DA, et al. Low literacy is associated with increased risk of hospitalization and death among individuals with heart failure. *J Gen Intern Med.* Sep 2013;28(9):1174-1180.
106. McNaughton CD, Collins SP, Kripalani S, et al. Low numeracy is associated with increased odds of 30-day emergency department or hospital recidivism for patients with acute heart failure. *Circ Heart Fail.* Jan 2013;6(1):40-46.
107. Murray MD, Tu W, Wu J, Morrow D, Smith F, Brater DC. Factors associated with exacerbation of heart failure include treatment adherence and health literacy skills. *Clin Pharmacol Ther.* Jun 2009;85(6):651-658.
108. Noureldin M, Plake KS, Morrow DG, Tu W, Wu J, Murray MD. Effect of health literacy on drug adherence in patients with heart failure. *Pharmacotherapy.* Sep 2012;32(9):819-826.
109. Macabasco-O'Connell A, DeWalt DA, Broucksou KA, et al. Relationship between literacy, knowledge, self-care behaviors, and heart failure-related quality of life among patients with heart failure. *J Gen Intern Med.* Sep 2011;26(9):979-986.
110. Peterson PN, Shetterly SM, Clarke CL, et al. Health literacy and outcomes among patients with heart failure. *JAMA.* Apr 27 2011;305(16):1695-1701.
111. Evangelista LS, Rasmusson KD, Laramee AS, et al. Health literacy and the patient with heart failure--implications for patient care and research: a consensus statement of the Heart Failure Society of America. *J Card Fail.* Jan 2010;16(1):9-16.
112. DeWalt DA, Schillinger D, Ruo B, et al. Multisite randomized trial of a single-session versus multisession literacy-sensitive self-care intervention for patients with heart failure. *Circulation.* Jun 2012;125(23):2854-2862.
113. Baker DW, Dewalt DA, Schillinger D, et al. The effect of progressive, reinforcing telephone education and counseling versus brief educational intervention on knowledge, self-care behaviors and heart failure symptoms. *J Card Fail.* Oct 2011;17(10):789-796.

114. Wu JR, Moser DK, Lennie TA, Peden AR, Chen YC, Heo S. Factors influencing medication adherence in patients with heart failure. *Heart Lung.* Jan-Feb 2008;37(1):8-16, 16 e11.
115. Jaarsma T, Beattie JM, Ryder M, et al. Palliative care in heart failure: a position statement from the palliative care workshop of the Heart Failure Association of the European Society of Cardiology. *Eur J Heart Fail.* May 2009;11(5):433-443.
116. Agren S, Evangelista L, Stromberg A. Do partners of patients with chronic heart failure experience caregiver burden? *European journal of cardiovascular nursing : journal of the Working Group on Cardiovascular Nursing of the European Society of Cardiology.* Dec 2010;9(4):254-262.
117. Pinquart M, Sörensen S. Correlates of physical health of informal caregivers: a meta-analysis. *J Gerontol B Psychol Sci Soc Sci.* Mar 2007;62(2):P126-137.
118. Perkins M, Howard VJ, Wadley VG, et al. Caregiving strain and all-cause mortality: evidence from the REGARDS study. *J Gerontol B Psychol Sci Soc Sci.* Jul 2013;68(4):504-512.
119. Ji J, Zöller B, Sundquist K, Sundquist J. Increased risks of coronary heart disease and stroke among spousal caregivers of cancer patients. *Circulation.* Apr 2012;125(14):1742-1747.
120. Candy B, Jones L, Drake R, Leurent B, King M. Interventions for supporting informal caregivers of patients in the terminal phase of a disease. *Cochrane Database Syst Rev.* 2011(6):CD007617.
121. Hodges LJ, Humphris GM, Macfarlane G. A meta-analytic investigation of the relationship between the psychological distress of cancer patients and their carers. *Soc Sci Med.* Jan 2005;60(1):1-12.
122. Stamataki Z, Ellis JE, Costello J, Fielding J, Burns M, Molassiotis A. Chronicles of informal caregiving in cancer: using 'The Cancer Family Caregiving Experience' model as an explanatory framework. *Support Care Cancer.* Feb 2014;22(2):435-444.
123. Wadhwa D, Burman D, Swami N, Rodin G, Lo C, Zimmermann C. Quality of life and mental health in caregivers of outpatients with advanced cancer. *Psychooncology.* Feb 2013;22(2):403-410.
124. Schulz R. Research priorities in geriatric palliative care: informal caregiving. *J Palliat Med.* Sep 2013;16(9):1008-1012.
125. Dunbar SB, Clark PC, Deaton C, Smith AL, De AK, O'Brien MC. Family education and support interventions in heart failure: a pilot study. *Nurs Res.* May-Jun 2005;54(3):158-166.
126. McMillan SC, Small BJ. Using the COPE intervention for family caregivers to improve symptoms of hospice homecare patients: a clinical trial. *Oncol Nurs Forum.* Mar 2007;34(2):313-321.
127. McMillan SC, Small BJ, Weitzner M, et al. Impact of coping skills intervention with family caregivers of hospice patients with cancer. *Cancer* 2005;106(1):214-222.
128. Susan C. McMillan BJSMWRSMTLMWEH. Impact of coping skills intervention with family caregivers of hospice patients with cancer. Vol 1062006:214-222.
129. McMillan SC, Small BJ, Haley WE, Zambroski C, Buck HG. The COPE Intervention for Caregivers of Patients with Heart Failure: An Adapted Intervention. *Journal of hospice and palliative nursing : JHPN : the official journal of the Hospice and Palliative Nurses Association.* Jun 1 2013;15(4).
130. Buck HG, Zambroski CH, Garrison C, McMillan SC. "Everything They Were Discussing, We Were Already Doing": Hospice Heart Failure Caregivers Reflect on a Palliative Caregiving Intervention. *Journal of hospice and palliative nursing : JHPN : the official journal of the Hospice and Palliative Nurses Association.* Jun 1 2013;15(4):218-224.
131. Riegel B, Lee CS, Dickson VV, Carlson B. An update on the self-care of heart failure index. *J Cardiovasc Nurs.* Nov-Dec 2009;24(6):485-497.
132. Riegel B, Lee CS, Sochalski J. Developing an instrument to measure heart failure disease management program intensity and complexity. *Circ Cardiovasc Qual Outcomes.* May 2010;3(3):324-330.
133. Riegel B, Moser DK, Anker SD, et al. State of the science: promoting self-care in persons with heart failure: a scientific statement from the American Heart Association. *Circulation.* Sep 22 2009;120(12):1141-1163.
134. Wagner E. Chronic disease management: what will it take to improve care for chronic illness? . *Effective Clinical Practice.* 1998;1:2-4.
135. Wagner EH, Austin BT, C. D, Hindmarsh M, Schaefer J, Bonomi A. Improving chronic illness care: translating evidence into action. *Health Aff.* 2001;20(6):64-78.
136. Wasson J, Splaine M, Bazos D, Fisher E. Working inside, outside, and side by side to improve the quality of health care. *Jt Comm J Qual Improv.* 1998;24:513-517.

137. Peikes D, Chen A, Schore J, Brown R. Effects of Care Coordination on Hospitalization, Quality of Care, and Health Care Expenditures Among Medicare Beneficiaries: 15 Randomized Trials. *JAMA : the journal of the American Medical Association.* February 11, 2009 2009;301(6):603-618.
138. Glasgow R, Whitesides H, Nelson C, King D. Use of the Patient Assessment of Chronic Illness Care (PACIC) with diabetic patients. *Diabetes Care.* 2005;28(11):2655-2661.
139. Wright A, Zhang B, Ray A, et al. Associations between end-of-life discussions, patient mental health, medical care near death, and caregiver bereavement adjustment. *JAMA : the journal of the American Medical Association.* 2008;300(14):1665-1673.
140. Danis M, Southerland LI, Garrett JM, et al. A prospective study of advance directives for life-sustaining care. *N Engl J Med.* Mar 28 1991;324(13):882-888.
141. Temel JS, McCannon J, Greer JA, et al. Aggressiveness of care in a prospective cohort of patients with advanced NSCLC. *Cancer.* 2008;113:826-833.
142. Teno J, Gruneir A, Schwartz Z, Nanda A, Welte T. Association between advance directives and quality of end-of-life care: A national study. *Journal of the American Geriatrics Society.* 2007;55:189-194.
143. Teno JM, Shu JE, Casarett D, Spence C, Rhodes R, Connor S. Timing of Referral to Hospice and Quality of Care: Length of Stay and Bereaved Family Members' Perceptions of the Timing of Hospice Referral. *Journal of pain and symptom management.* 2007/8 2007;34(2):120-125.
144. Meyers F, Linder J. Simultaneous care: Disease treatment and palliative care throughout illness. *Journal of Clinical Oncology.* 2003;21(7):1412-1415.
145. Earle CC, Landrum MB, Souza JM, Neville BA, Weeks JC, Ayanian JZ. Aggressiveness of cancer care near the end of life: is it a quality-of-care issue? *Journal of clinical oncology : official journal of the American Society of Clinical Oncology.* Aug 10 2008;26(23):3860-3866.
146. Berkman LF, Blumenthal J, Burg M, et al. Effects of treating depression and low perceived social support on clinical events after myocardial infarction: the Enhancing Recovery in Coronary Heart Disease Patients (ENRICHD) Randomized Trial. *JAMA.* Jun 18 2003;289(23):3106-3116.
147. Czajkowski S, Arteaga S, Burg M, R A, Fisher J. Social support and coronary artery disease. In: Allan R, Fisher J, eds. *Heart and mind: The practice of cardiac psychology*. 2nded. Washington, DC: American Psychological Association; 2011:169-195.

148. Lett HS, Blumenthal JA, Babyak MA, Strauman TJ, Robins C, Sherwood A. Social support and coronary heart disease: epidemiologic evidence and implications for treatment. *Psychosom Med.* Nov-Dec 2005;67(6):869-878.
149. Brink E, Karlson BW, Hallberg LRM. Health experiences of first-time myocardial infarction: factors influencing women's and men's health-related quality of life after five months. *Psychology, Health & Medicine.* 2002;7(1):5-16.
150. Brown JR, Gillinder B. The relationship between coping style and affect in recovering cardiac patients. *Current Research in Social Psychology.* 2001;6(11):151-165

151. van Elderen T, Maes S, Dusseldorp E. Coping with coronary heart disease: a longitudinal study. *J Psychosom Res.* Aug 1999;47(2):175-183.
152. O'Hara RE, Hull JG, Lyons KD, et al. Impact on caregiver burden of a patient-focused palliative care intervention for patients with advanced cancer. *Palliat Support Care.* Dec 2010;8(4):395-404.
153. Williams AW, Bakitas M. Cancer Family Caregivers: A New Direction for Interventions. *J Palliative Med. 15(7): 775-783. PMID: 22612407; PMCID: PMC3387766*
154. Williams AW, Bakitas M, Prigerson H. Determinants of Prolonged Grief in Cancer Family Caregivers. National Palliative Care Research Center Junior Career Development Award; 2011-2013.
155. Miaskowski C. Symptom Trajectories, Palliative Care, and Quality of Life. Paper presented at: The Science of Compassion: Future Directions in End-of-Life and Palliative Care2011; Bethesda, MD.
156. Miaskowski C, Dhruva A, Dodd M, et al. Trajectories of Fatigue in Patients With Breast Cancer Before, During, and After Radiation Therapy. *Cancer nursing.* May-Jun 2010;33(3):201-212.
157. Ritchie CS, Currow DC, Abernethy AP, Kutner JS. Multisite studies offer a solution to recruitment challenges in palliative care studies. *J Palliat Med.* Mar 2013;16(3):225.
158. Lu H, Trancik E, Bailey FA, et al. Families' perceptions of end-of-life care in Veterans Affairs versus non-Veterans Affairs facilities. *J Palliat Med.* Aug 2010;13(8):991-996.
159. Kvale E, Ritchie C, Sudenga S, Jackson M, Kno H, Meleth S. Comorbidity Does Not Predict Symptom Burden in Palliative Care Outpatient (730). *J Pain Symptom Manag.* Feb 2010;39(2):438-438.

160. Kvale E, Ekundayo O, Ritchie C, Ahmed A. Predictors of Mortality among Community-Dwelling Older Adults with a History of Cancer in a Prospective Population-Based Study. *Gerontologist.* Oct 2008;48:414-414.
161. Casarett D, Pickard A, Bailey FA, et al. A nationwide VA palliative care quality measure: the family assessment of treatment at the end of life. *J Palliat Med.* Jan-Feb 2008;11(1):68-75.
162. Patsopoulos NA. A pragmatic view on pragmatic trials. *Dialogues in clinical neuroscience.* 2011;13(2):217-224.
163. Thorpe KE, Zwarenstein M, Oxman AD, et al. A pragmatic-explanatory continuum indicator summary (PRECIS): a tool to help trial designers. *J Clin Epidemiol.* May 2009;62(5):464-475.
164. Falk H, Ekman I, Anderson R, Fu M, Granger B. Older Patients' Experiences of Heart Failure—An Integrative Literature Review. *Journal of Nursing Scholarship.* 2013;45(3):247-255.
165. Kurland BF, Johnson LL, Egleston BL, Diehr PH. Longitudinal Data with Follow-up Truncated by Death: Match the Analysis Method to Research Aims. *Stat Sci.* 2009;24(2):211.
166. Byock I. Palliative Care and Oncology: Growing Better Together. *J Clin Oncol.* January 10, 2009 2009;27(2):170-171.
167. Bakitas M, Tosteson TD, Li Z, et al. The ENABLE III Randomized Controlled Trial of Concurrent Palliative Oncology Care: Does Timing Matter? *JAMA : the journal of the American Medical Association.* 2014, under review.
168. Dionne-Odom JN, Tosteson TD, Li Z, et al. The ENABLE III Randomized Controlled Trial of Concurrent Palliative Oncology Care: Caregiver Benefits. *JAMA : the journal of the American Medical Association.* 2014, under review.
169. Temel J, Jackson V, Billings A, et al. Phase II Study: Integrated palliative care in newly diagnosed advanced non-small cell lung cancer patients. *J Clin Oncol.* 2007;25(17):2377-2382.
170. Follwell M, Burman D, Le LW, et al. Phase II Study of an Outpatient Palliative Care Intervention in Patients With Metastatic Cancer. *Journal of clinical oncology : official journal of the American Society of Clinical Oncology.* January 10, 2009 2009;27(2):206-213.
171. McNiff KK, Neuss MN, Jacobson JO, Eisenberg PD, Kadlubek P, Simone JV. Measuring supportive care in medical oncology practice: lessons learned from the quality oncology practice initiative. *J Clin Oncol.* Aug 10 2008;26(23):3832-3837.
172. National Comprehensive Cancer Network. *Palliative Care V.I.2006, NCCN Clinical Practice Guidelines in Oncology.* New York: National Comprehensive Cancer Network;2006.
173. National Quality Forum. *A National Framework and Preferred Practices for Palliative and Hospice Care Quality: A Consensus Report.* Washington, DC: National Quality Forum;2006.
174. Bakitas M, Macmartin M, Trzepkowski K, et al. Palliative care consultations for heart failure patients: how many, when, and why? *J Card Fail.* Mar 2013;19(3):193-201.
175. Dionne-Odom JN, Kono A, Frost J, et al. Translating and Testing the ENABLE CHF PC Concurrent Palliative Care Model for Older Adults with Heart Failure and Their Family Caregivers. *Journal of palliative medicine.* 2014, in REVISION/RESUBMISSION.
176. Shuaib FM, Durant RW, Parmar G, et al. Awareness, treatment and control of hypertension, diabetes and hyperlipidemia and area-level mortality regions in the Reasons for Geographic and Racial Differences in Stroke (REGARDS) study. *Journal of health care for the poor and underserved.* May 2012;23(2):903-921.
177. Safford MM, Brown TM, Muntner PM, et al. Association of race and sex with risk of incident acute coronary heart disease events. *JAMA.* Nov 7 2012;308(17):1768-1774.
178. Durant RW, Parmar G, Shuaib F, et al. Awareness and management of chronic disease, insurance status, and health professional shortage areas in the REasons for Geographic And Racial Differences in Stroke (REGARDS): a cross-sectional study. *BMC health services research.* 2012;12:208.
179. Durant RW, Legedza AT, Marcantonio ER, Freeman MB, Landon BE. Different types of distrust in clinical research among whites and African Americans. *Journal of the National Medical Association.* Feb 2011;103(2):123-130.
180. Durant RW, Legedza AT, Marcantonio ER, Freeman MB, Landon BE. Willingness to participate in clinical trials among African Americans and whites previously exposed to clinical research. *Journal of cultural diversity.* Spring 2011;18(1):8-19.
181. Bailey FA, Williams BR, Goode PS, et al. Impact of a hospice emergency kit for veterans and their caregivers: A prospective cohort study. *journal of Hospice and Palliative Medicine.* 2014, in press.
182. WC. L. Seattle Heart Failure Model. *The American Journal of Cardiology.* 2013;111(8):1235.

183. Callahan C, Unverzagt F, Hui S, Perkins A, Hendrie H. Six-item screener to identify cognitive impairment among potential research subjects. *Medical Care.* 2002;40(9):771-781.
184. Dunbar SB, Clark PC, Quinn C, Gary RA, Kaslow NJ. Family influences on heart failure self-care and outcomes. *J Cardiovasc Nurs.* May-Jun 2008;23(3):258-265.
185. Bakitas M. Insights from a Family Focus Group on Hospital End-of-Life Care1999. Located at: Unpublished data, Dartmouth-Hitchcock Medical Center.
186. Bakitas M, Daretany K. Improving End-of-Life Care: Hospital-based Palliative Care. In: Ferrell B, Coyle N, eds. *Oxford Textbook of Palliative Nursing, 2nd Edition.* Oxford: Oxford University Press; 2006:787-815.
187. Bakitas MA. Background Noise: The experience of chemotherapy-induced peripheral neuropathy. *Nursing research.* 2007;56(5):323-331.
188. Skalla K, Bakitas M, Furstenberg C, Ahles T, Henderson J. Patients' need for information about cancer therapy. *Oncol Nurs Forum.* 2004;31(2):313-320.
189. Bakitas M, Lyons Doyle K. Oncology Clincians' Perspectives on Providing Palliative Care for their Patients with Advanced Cancer. Paper presented at: 10th National Conference on Cancer Nursing Research2009; Orlando, FL
190. Flemming K, Adamson J, Atkin K. Improving the effectiveness of interventions in palliative care: the potential role of qualitative research in enhancing evidence from randomized controlled trials. *Palliative medicine.* Mar 2008;22(2):123-131.
191. Protheroe J, Bower P, Chew-Graham C. The use of mixed methodology in evaluating complex interventions: identifying patient factors that moderate the effects of a decision aid. *Fam. Pract.* December 1, 2007 2007;24(6):594-600.
192. Lyons KD, Bakitas M, Hegel MT, Hanscom B, Hull J, Ahles TA. Reliability and validity of the Functional Assessment of Chronic Illness Therapy-Palliative care (FACIT-Pal) scale. *J Pain Symptom Manage.* Jan 2009;37(1):23-32.
193. Green CP, Porter CB, Bresnahan DR, Spertus JA. Development and evaluation of the Kansas City Cardiomyopathy Questionnaire: a new health status measure for heart failure. *Journal of the American College of Cardiology.* Apr 2000;35(5):1245-1255.
194. Bjelland I, Dahl AA, Haug TT, Neckelmann D. The validity of the Hospital Anxiety and Depression Scale: An updated literature review. *Journal of Psychosomatic Research.* 2002;52(2):69-77.
195. Weitzner M, Jacobsen, P.B., Wagner, H., Friedland, J., & Cox, C. The caregiver quality of life index-cancer (CQOL-C) scale: Development and validation of an instrument to measure quality of life of the family of caregiver of patients with cancer. *Quality of Life Research* 1999;8:55-63.
196. Montgomery RJV, Borgatta EF, Borgatta ML. Societal and family change in the burden of care. In: Liu WT, Kendig H, eds. *Who Should Care for the Elderly?* Singapore: Singapore University Press; 2000:27-54.
197. Rosemann T, Laux G, Szecsenyi J, Grol R. The Chronic Care Model: congruency and predictors among primary care patients with osteoarthritis. *Qual Saf Health Care.* Dec 2008;17(6):442-446.
198. Carver CS. You want to measure coping but your protocol's too long: Consider the brief COPE. *International Journal of Behavioral Medicine.* 1997;4:92-100.
199. Oxman TE, Hull JG. Social support and treatment response in older depressed primary care patients. *J Gerontol B Psychol Sci Soc Sci.* Jan 2001;56(1):P35-45.
200. Bakitas M, Ahles T, Skalla K, et al. Proxy perspectives regarding end-of-life care for persons with cancer. *Cancer.* 2008;112(8):1854-1861.
201. Downey L, Curtis JR, Lafferty WE, Herting JR, Engelberg RA. The Quality of Dying and Death Questionnaire (QODD): empirical domains and theoretical perspectives. *J Pain Symptom Manage.* Jan 2010;39(1):9-22.
202. Curtis JR, Patrick DL, Engelberg RA, Norris K, Asp C, Byock I. A measure of the quality of dying and death. Initial validation using after-death interviews with family members. *J Pain Symptom Manage.* Jul 2002;24(1):17-31.
203. Prigerson HG, Maciejewski PK, Reynolds C, et al. Inventory of Complicated Grief: A scale to measure maladaptive symptoms of loss. *Psychiatry Research.* 1995;59:65-79.
204. MacKinnon DP. *Introduction to statistical mediation analysis.* New York: Taylor and Francis; 2008.
205. MacCallum R, Browne M, Sugawara H. Power analysis and determination of sample size for covariance structure modeling. *Psychological Methods.* 1996;1(2):130-149.

206. Fritz MS, Mackinnon DP. Required sample size to detect the mediated effect. *Psychol Sci.* Mar 2007;18(3):233-239.
207. Garin O, Ferrer M, Pont Ä, et al. Disease-specific health-related quality of life questionnaires for heart failure: a systematic review with meta-analyses. *Quality of Life Research.* 2009;18(1):71-85.
208. Sullivan MD, Newton K, Hecht J, Russo JE, Spertus JA. Depression and health status in elderly patients with heart failure: a 6-month prospective study in primary care. *The American journal of geriatric cardiology.* Sep-Oct 2004;13(5):252-260.
209. Pedersen SS, Hoogwegt MT, Jordaens L, Theuns DAMJ. Relation of Symptomatic Heart Failure and Psychological Status to Persistent Depression in Patients With Implantable Cardioverter-Defibrillator. *The American journal of cardiology.* 2011;108(1):69-74.
