# Supplementary material for: Effects of a Telehealth Early Palliative Care Intervention for Family Caregivers of Persons With Advanced Heart Failure: The ENABLE CHF-PC Randomized Clinical Trial
Source: JAMA Netw Open. 2020 Apr 13;3(4):e202583. doi: 10.1001/jamanetworkopen.2020.2583 (PMC7154802; doi:10.1001/jamanetworkopen.2020.2583)
Supplement: Supplement 2. — Data Sharing Statement [file jamanetwopen-3-e202583-s002.pdf]

Dionne-Odom. Effects of a telehealth early palliative care intervention vs usual care for family caregivers of persons with advanced heart failure. *JAMA Netw Open*. 2020;3(4):e202583. 10.1001/jamanetworkopen.2020.2583

Data Sharing Statement

Data

Data available: No
